# HPD is an m^6^A Methyltransferase that Protects Colorectal Cancer Cells from Ferroptotic Cell Death by m^6^A Methylating SLC7A11/GPX4

**DOI:** 10.1002/advs.202508541

**Published:** 2025-11-29

**Authors:** Jiyan Wang, Xintong Dai, Huanle Liu, Saiwei Hua, Hongkai Chang, Huanran Sun, Mingming Sun, Huifang Zhao, Kemin Ni, Fei Xie, Yaya Qiao, Qingle Gao, Chenxi Yu, Qijun Zhang, Jianshuang Guo, Chunze Zhang, Shuai Zhang, Changliang Shan

**Affiliations:** ^1^ State Key Laboratory of Medicinal Chemical Biology College of Pharmacy and Tianjin Key Laboratory of Molecular Drug Research Nankai University Tianjin 300350 China; ^2^ School of Integrative Medicine State Key Laboratory of Chinese Medicine Modernization Tianjin University of Traditional Chinese Medicine Tianjin 301617 China; ^3^ School of Medicine Nankai University Tianjin 300071 China; ^4^ College of Pharmacy Hebei University Baoding 071002 China; ^5^ Department of Colorectal Surgery Tianjin Union Medical Center Tianjin 300121 China

**Keywords:** colorectal cancer, ferroptosis, HPD, m^6^A modification

## Abstract

N6‐methyladenosine (m^6^A) is a dynamic RNA modification, which is added by the METTL3‐METTL14 methyltransferase complex or METTL16. Unexpectedly, the tyrosine metabolism enzyme 4‐hydroxyphenylpyruvate dioxygenase (HPD) is discovered as a methyltransferase responsible for m^6^A modification. Unlike METTL3, which requires the assistance of METTL14 to form a complex and exert methyltransferase activity. Interestingly, it is revealed that HPD has a catalytic domain (CMI) like METTL3. Moreover, HPD recruits the universal cofactor S‐adenosylmethionine (SAM) to the substrate binding center as a methyl group donor. In particular, it is demonstrated that HPD regulates colorectal cancer ferroptosis by methylating SLC7A11/GPX4 through a moonlighting function. These findings uncover the moonlighting function of HPD in m^6^A‐mediated ferroptosis and underscore the potential to target the m^6^A methyltransferase activity of HPD for cancer treatment.

## Graphical abstract

This study reveals that the tyrosine metabolic enzyme HPD functions as a previously uncharacterized, METTL3‐independent m^6^A methyltransferase. It promotes colorectal tumor progression by coordinately regulating the SLC7A11/GPX4 axis to suppress ferroptosis. This finding expands the family of m^6^A writers and reveals a promising new target for therapeutic intervention.

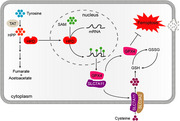

## 1 Introduction

N6‐methyladenosine (m^6^A) modification is the most abundant chemical modification of messenger RNAs (mRNAs), which exquisitely and plastically regulates gene expression and biological processes.^[^ ^1^, ^2^ ^]^ N6‐methyladenosine modification is added by methyltransferases (writers), removed by demethylases (erasers), and recognized by readers. Methyltransferase are currently known to function as a complex that includes METTL3, METTL14, WTAP, VIRMA, RBM15/15B, HAKAI, and ZC3H133. The METTL3 protein is the catalytic subunit, and other component proteins are responsible for coordinating the activity of METTL3 and binding RNA.^[^ ^3^ ^]^ There still exists m^6^A modification in METTL3 knockdown cells,^[^ ^4^, ^5^ ^]^ which means that there is also a portion of m^6^A modification that is not catalyzed by METTL3. These results indicate that there may be other methyltransferase proteins, which are responsible for m^6^A modification. Although METTL16 has also been shown to possess methyltransferase activity, its substrates are pre‐mRNA and non‐coding RNA.^[^ ^6^ ^]^ Therefore, the identification of novel mRNA m^6^A methyltransferases is of great significance for exploring the biogenesis of m^6^A modification.

Reprogramming of tyrosine metabolism has been shown to play an important role in tumorigenesis and progression.^[^ ^7^, ^8^, ^9^, ^10^, ^11^ ^]^ Recently, our results show that HPD is highly expressed in breast and lung cancer.^[^ ^7^, ^12^ ^]^ Furthermore, we unveiled that HPD coordinates the tyrosine metabolism pathway and anabolic biosynthesis mediated by the AMPK‐HDAC10‐G6PD axis.^[^ ^7^ ^]^ An increasing number of metabolic enzymes have been shown to have both non‐enzymatic and multi‐enzymatic activities.^[^ ^13^, ^14^, ^15^ ^]^ However, whether HPD has multi‐enzymatic activities in regulating cancer development has not been reported. In this study, we made the striking discovery that HPD is the m^6^A methyltransferase for mRNA.

In the current study, we surprisingly demonstrate that HPD is specifically localized in the nucleus and acts as an m^6^A methyltransferase to promote colorectal cancer progression by protecting cells from DNA damage and ferroptotic death. Collectively, our results demonstrate that the metabolic enzyme HPD acts as an m^6^A methyltransferase, which adds another dimension to the regulation of m^6^A modification beyond that by the METTL3‐METTL14 methyltransferase complex or METTL16.

## 2 Results

### 2.1 HPD is Located in the Nucleus and Regulates the m^6^A Modification

**Figure 1:**
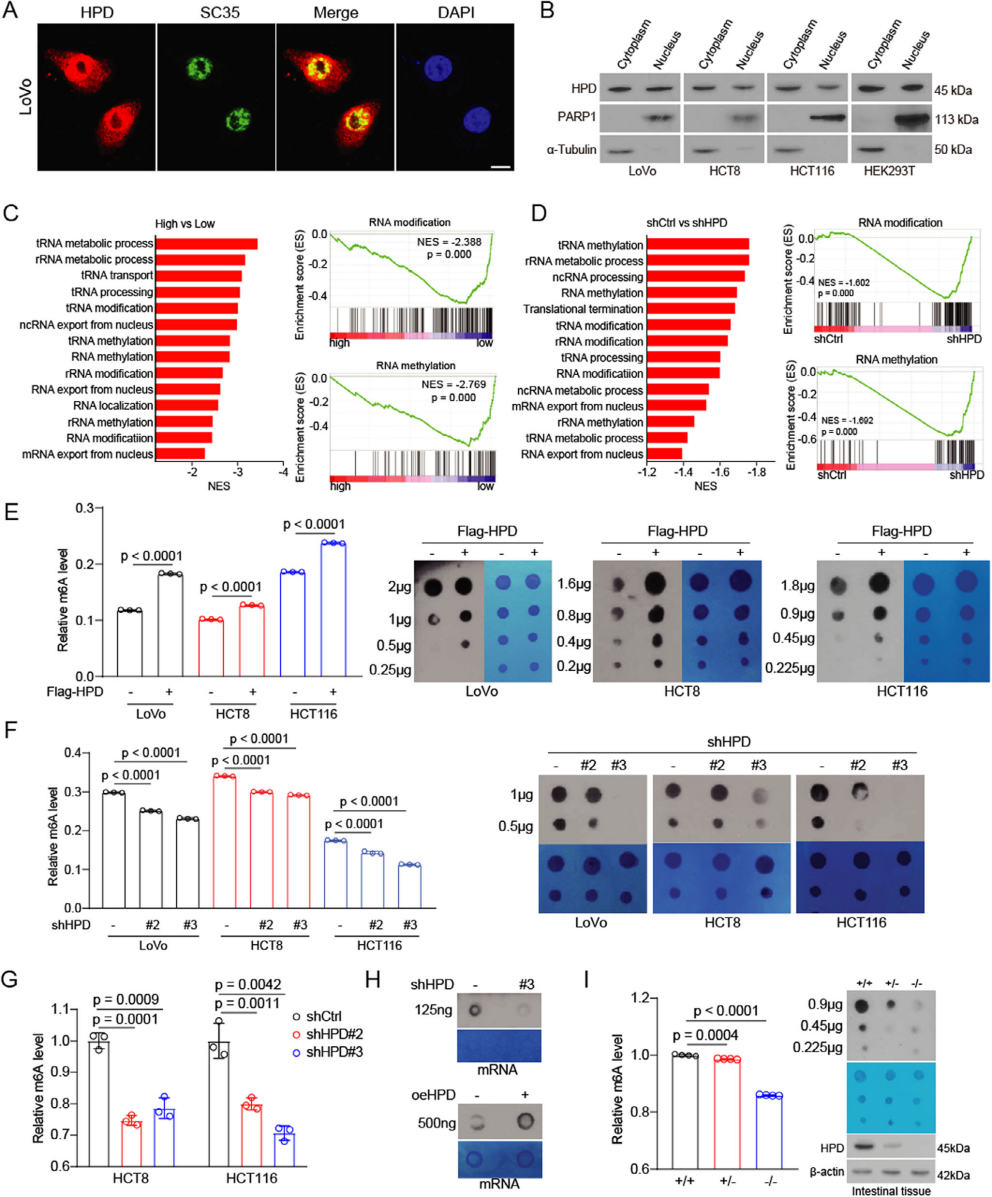
HPD localizes to the nucleus and regulates m^6^A modification. A) LoVo cells were stained for HPD and SC35 via cell immunofluorescence. Scar bars, 10 µm. B) The location of HPD was detected in different cells. C–D) The pathways associated with HPD were analyzed by gene set enrichment analysis (GSEA) in the TCGA database and through RNA‐seq. (shCtrl, *n* = 3; shHPD, *n* = 3). E) The m^6^A modification of total RNA was detected in tumor cells with overexpression HPD by colorimetric assay and dot blot. (Control, *n* = 3; Flag‐HPD, *n* = 3). F) The m^6^A modification of total RNA was detected in HPD knockdown cells by colorimetric assay and dot blot. (shCtrl, *n* = 3; shHPD #2, *n* = 3; shHPD #3, *n* = 3). G) The m^6^A modification of mRNA was detected in HPD knockdown cells by LC/MS. (shCtrl, *n* = 3; shHPD #2, *n* = 3; shHPD #3, *n* = 3). H) The m^6^A modification of mRNA was detected in HPD knockdown or HPD overexpressing cells by dot blot. I) The m^6^A modification of total RNA was detected in HPD knockout mice by colorimetric assay and dot blot. (+/+, *n* = 4; +/‐, *n* = 4; ‐/‐, *n* = 4). Error bars in E, F, G, and I, represent mean values ± SD, *p* values were determined by an unpaired two‐tailed Student's t test of technical replicates.

The tyrosine metabolic process is usually thought to occur in the cytoplasm. Here, we strikingly discover that HPD exhibits a nuclear speckles pattern in different cells (Figure S1A, Supporting Information). To clarify the cellular localization of HPD, we performed immunofluorescence and nucleoplasm separation experiments and found that HPD localizes in both the nucleus and cytoplasm in colorectal cancer cells and HEK293T cells (**Figure** **1**A,B). Interestingly, HPD has been shown to co‐localize well with mRNA‐processing factors (serine/arginine‐rich splicing factor 2, SRSF2/SC35) in nuclear speckles. These results suggest that HPD may have potential regulation on mRNA in the nucleus.

To investigate whether and how HPD functions on mRNA inside the nucleus, we analyzed possible biological processes based on HPD expression in the colorectal cancer TCGA database. We found that RNA modification, RNA methylation, and RNA processing were enriched (Figure 1C), which suggests that HPD functions on RNA modification. To validate the above results, we performed transcriptome‐wide RNA‐sequencing and got similar results (Figure 1D; Figure S1B, Supporting Information). When we treated the cells with RNase A, the HPD signal within the nucleus was significantly reduced, indicating a cellular interaction between HPD and nuclear RNA (Figure S1C, Supporting Information). In short, our findings suggest the potential regulation of HPD on RNA modification. Our previous research identified HPD as an RNA‐binding protein that specifically binds to the coding sequence (CDS) of mRNA via a motif corresponding to the m^6^A consensus.^[^ ^16^ ^]^ Therefore, in this study, our initial objective is to evaluate the role of HPD in the m^6^A methylation of mRNA.

To validate whether HPD functions as a regulator of mRNA m^6^A modification, we examined the effect of HPD on m^6^A modification. Indeed, we found that the m^6^A modification of total RNA significantly increased, as determined by dot blot and m^6^A quantification assay, in cells with exogenous HPD expression (Figure 1E). Conversely, the m^6^A modification is significantly decreased in HPD knocked down cells (Figure 1F). We also employed liquid chromatography‐mass spectrometry (LC/MS) to confirm that knocking down HPD decreased m^6^A modification at the mRNA level (Figure 1G). The m^6^A modification is almost distributed across all types of RNA, especially mRNA (1). The results showed that HPD regulates the m^6^A modification of mRNA (Figure 1H). Moreover, a significant reduction in m^6^A modification was also observed in the intestinal tissues of the HPD knockout mouse model (Figure 1I). These results imply that HPD has a positively regulates m^6^A modification.

### 2.2 HPD Directly Catalyze m^6^A Modification in an METTL3‐Independent Manner

**Figure 2:**
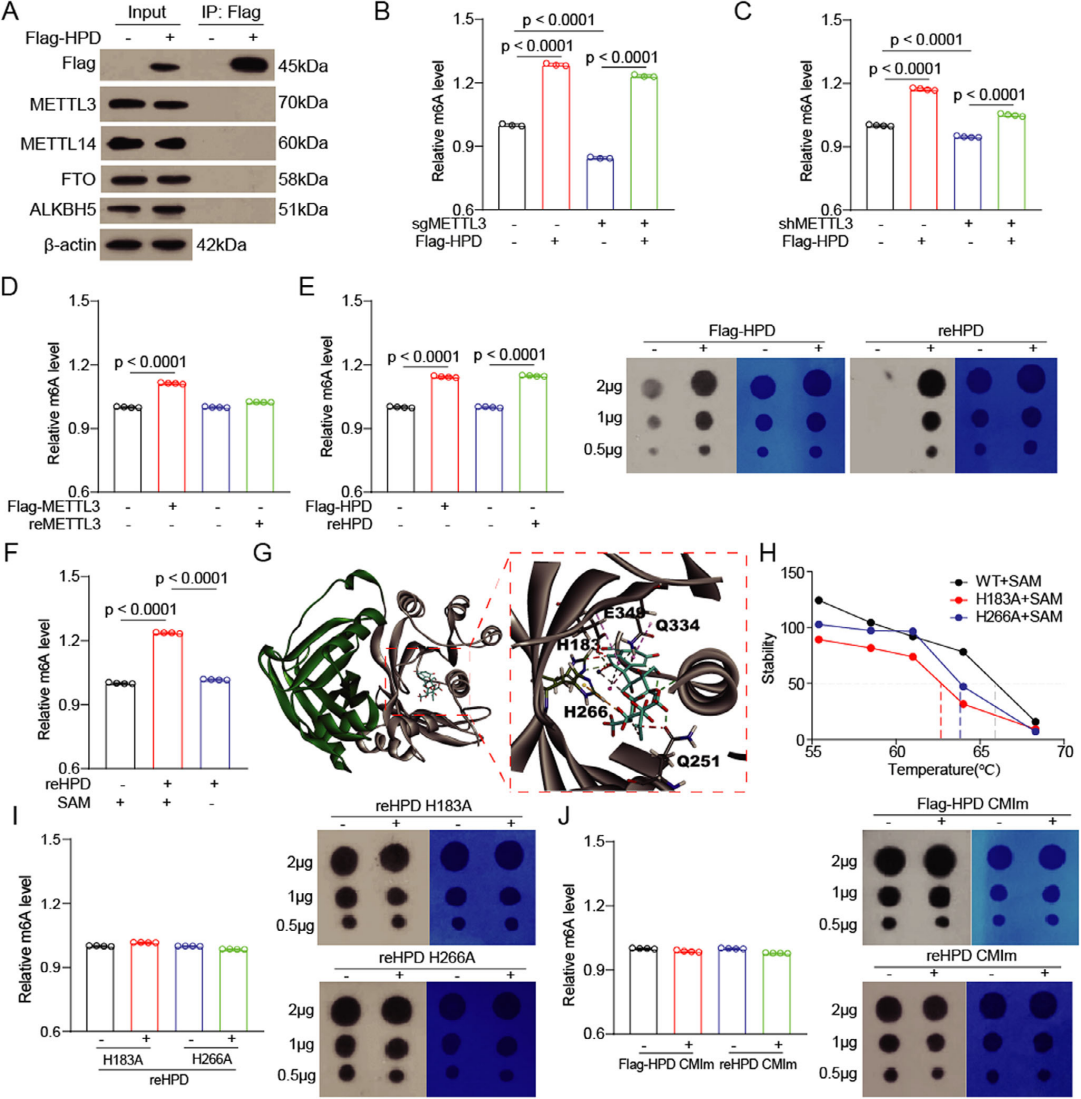
HPD acts as an methyltransferase to catalyze RNA methylation modification in vitro. A) The interaction between HPD and m^6^A‐related proteins was detected by co ‐immunoprecipitation assay in HPD overexpressing cells. B–C) The m^6^A modification of total RNA was detected in HCT8 cells with METTL3 knockout/knockdown and re‐overexpressed HPD by colorimetric assay. (B, *n* = 3; C, *n* = 4). D–E) The in vitro methylation assay was employed for assess the methyltransferase activity of Flag‐METTL3 and Flag‐HPD (from eukaryotic cells), reMETTL3 and reHPD (from *E.coli*), and the m^6^A modification of total RNA was detected by colorimetric assay and dot blot. F) The in vitro methylation assay was employed to assess the methyltransferase activity of reHPD with or without SAM. The m^6^A modification of total RNA was detected by colorimetric assay. G) Molecular docking analysis was conducted between protein HPD (PDB ID: 3ISQ) and SAM. H) The thermol shift assay was performed to detect the binding between HPD (wildtype and mutant) and SAM. I) The in vitro methylation assay was employed to assess the methyltransferase activity of reHPD (H183A and H266A). J) The in vitro methylation assay was employed to assess the methyltransferase activity of Flag‐HPD CMIm and reHPD CMIm and the m^6^A modification of total RNA was detected by colorimetric assay and dot blot. Error bars in B, C, D, E, F, I, and J, represent mean values ± SD, *p* values were determined by an unpaired two‐tailed Student's *t* test of *n* = 4 technical replicates.

As the m^6^A modification is added by the METTL3‐METTL14 complex and many proteins of the complex have been identified,^[^ ^3^, ^17^, ^18^ ^]^ we wondered whether HPD, as a component of the methyltransferase complex, positively regulates m^6^A modification. A Co‐immunoprecipitation assay showed that there is no interaction between HPD and METTL3 complex (**Figure** **2**A; Figure S2A,B, Supporting Information). And knockdown of HPD did not significantly alter METTL3 expression and methyltransferase activity (Figure S2C,D, Supporting Information). More importantly, HPD still increases m^6^A modification under METTL3 knockout and knockdown conditions (Figure 2B,C; Figure S2E,F, Supporting Information). These results suggest that HPD regulates m^6^A modification independently of the METTL3‐METTL14 complex. In other words, HPD may act as an m^6^A methyltransferase to generate m^6^A modification.

To investigate whether HPD functions as an m^6^A methyltransferase, we established an in vitro methylation assay by using Flag‐METTL3 from eukaryotic cells and the recombinant protein METTL3 (reMETTL3) from *E.coli*. The dot blot and m^6^A quantification assay results indicated that Flag‐METTL3, rather than reMETTL3, significantly increased the m^6^A modification (Figure 2D; Figure S2G, Supporting Information). METTL3 is merely the catalytic subunit in the m^6^A methyltransferase complex, and its activity necessitates the assistance of other proteins.^[^ ^19^ ^]^ There is a strong binding affinity between METTL3 and METTL14 (Figure S2B, Supporting Information), and it is probable that other complex proteins are present in the Flag‐METTL3 IP complex. However, reMETTL3 lacks m^6^A methyltransferase activity in the absence of accessory proteins. Meanwhile, we also excluded the possibility that the protein itself enhances the stability of RNA and results in an increase of m^6^A modification (Figure S2H, Supporting Information). These results confirm the effectiveness of the in vitro methylation assay we established.

Next, we detected the methyltransferase activity of HPD in vitro, and found that m^6^A modification was dramatically increased by Flag‐HPD or recombinant protein HPD (reHPD) (Figure 2E). Unlike METTL3, reHPD expressed from *E.coli* also has m^6^A methyltransferase activity, which means that HPD acts as an m^6^A methyltransferase and does not require other proteins. These results not only confirm that HPD is an m^6^A methyltransferase but also suggest that HPD exerts methyltransferase activity independently.

### 2.3 HPD Recruits S‐Adenosylmethionine as a Methyl Group Donor

S‐adenosylmethionine (SAM) is a common methyl donor in mammals and serves as the cofactor for METTL3 and METTL16.^[^ ^19^, ^20^ ^]^ Similar to METTL3, the methyltransferase activity of HPD was inhibited when SAM was removed from the reaction (Figure 2F; Figure S2I,J, Supporting Information). Moreover, under non‐reHPD conditions, SAM also failed to increase m^6^A modification (Figure S2K, Supporting Information).

To explore the binding sites of HPD and SAM, molecular docking based on the crystal structure of HPD (PDB ID: 3ISQ) was performed. It was found that SAM fits in a substrate (4‐hydrophenylpyruvate, HPP) pocket, surrounded by residues including H183, H266, Q251, Q334, and E349 of HPD (Figure 2G). Compared with tyrosine, SAM treatment also promoted the thermal stability of HPD (Figure S3A, Supporting Information). Then, based on molecular docking analysis, we selected H183 and H266 as candidate sites. The thermal shift assay showed that the mutation of H183 and H266 in HPD reduces the ability to bind to SAM (Figure 2H; Figure S3B, Supporting Information). The surface plasmon resonance (SPR) assay also showed the direct interaction between SAM and HPD, and mutations at H183 and H266 reduced the binding capacity between HPD and SAM (Figure S3C, Supporting Information). Finally, compared with wild‐type HPD, the mutant HPD (H183A and H266A) lost the ability to increase m^6^A modification (Figure 2I). These results suggested that the H183 and H266 sites mediate the binding of HPD and SAM.

### 2.4 Catalytic Motif (CMI) Mediates Methyltransferase Activity of HPD

Next, we aim to identify which catalytic site of HPD is responsible for the m^6^A modification on the mRNA. METTL3 has been reported to possess two conserved catalytic motifs (CM):^[^ ^21^ ^]^ CMI and CMII (Figure S3D, Supporting Information). Flag‐METTL3 lost its methylate ability when CMI and CMII were mutated (Figure S3E, Supporting Information). Through sequence alignment, we discovered that HPD has a motif similar to CMI, and CMI is highly conserved during evolution (Figure S3F, Supporting Information). When the CMI of HPD was mutated, HPD lost its m^6^A methyltransferase activity (Figure 2J). Obviously, HPD exerts m^6^A methyltransferase activity that is dependent on its catalytic motif (CMI).

### 2.5 Methyltransferase Activity and Tyrosine Metabolizing Enzyme Activity of HPD

**Figure 3:**
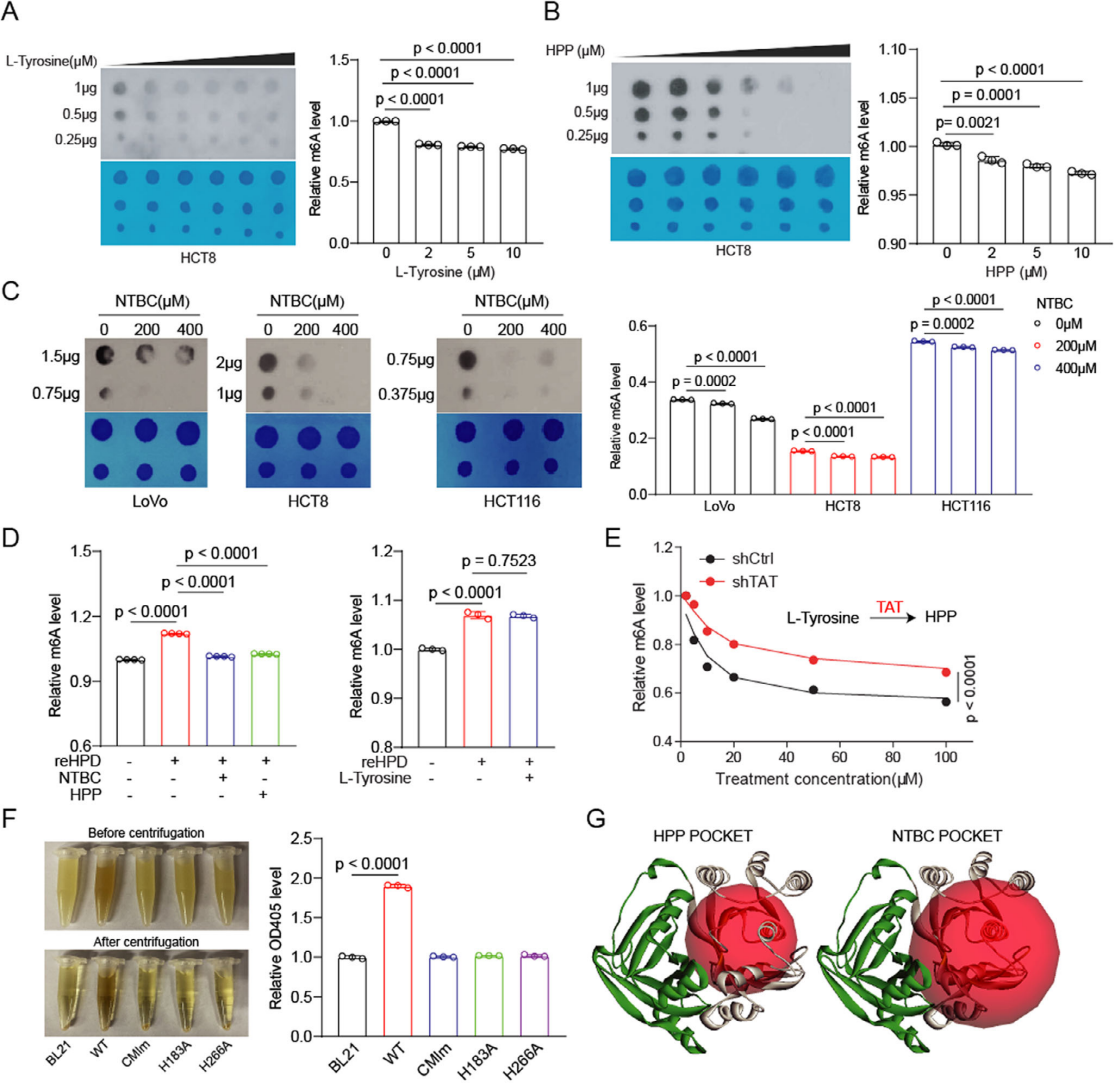
The methyltransferase activity and the tyrosine‐metabolizing enzyme activity of HPD share an activity pocket. A–B) The m^6^A modification of total RNA was detected in tyrosine or HPP treated cells by colorimetric assay and dot blot. C) The m^6^A modification of total RNA was detected in NTBC treated cells by colorimetric assay and dot blot. D) The in vitro methylation assay was employed to assess the methyltransferase activity of reHPD and different small molecules (HPP, NTBC and Tyrosine). The m^6^A modification of total RNA was detected by colorimetric assay. E) The m^6^A modification of total RNA was detected in HCT8 cells with knockdown TAT and treated with Tyrosine by colorimetric assay. F) The activity of the tyrosine‐metabolizing enzyme was measured using the bacterial metabolism assay. G) The active centers of HPD binding to HPP and NTBC were displayed. Error bars in A, B, C, D, and F, represent mean values ± SD, *p* values were determined by unpaired two‐tailed Student's *t* test of *n* = 3 or 4 technical replicates.

To further elucidate the association between the metabolic and methylation functions of HPD, we treated cells with HPD upstream metabolites (Tyrosine and HPP) and an HPD inhibitor (NTBC). The results showed that treatment with the metabolite and the inhibitor decreased the global m^6^A levels (**Figure** **3**A–C; Figure S4A, Supporting Information). Further results showed that metabolites and inhibitors did not alter METTL3 expression and methyltransferase activity (Figure S4B, Supporting Information). However, in HPD knockdown cells, the metabolites and inhibitors no longer have the ability to significantly modulate the level of m^6^A modification (Figure S4C, Supporting Information). These data suggest that both the metabolites and the inhibitors are targeted to HPD to regulate m^6^A modification. Interestingly, in vitro experiments demonstrated that NTBC and HPP, but not Tyrosine, inhibited its activity by directly binding to HPD (Figure 3D; Figure S4D, Supporting Information). Nitisinone (NTBC) is used to treat type III tyrosinemia by inhibiting tyrosine metabolism and also fits into a substrate (HPP) pocket.^[^ ^22^ ^]^ The ability of tyrosine to inhibit methylation modification was significantly weakened when the metabolic enzyme TAT, which converts tyrosine to HPP, was knocked down (Figure 3E; Figure S4E, Supporting Information). To further clarify the effect of tyrosine and HPP, we found that HPP, but not tyrosine, inhibited the methyltransferase activity of HPD using dose‐dependent treatment in in vitro methylation experiments (Figure S4F,G, Supporting Information). We also explored the effect of HPD mutations (CMI, H183, and H266) on tyrosine metabolism activity and showed that these mutants eliminated the tyrosine metabolism enzyme activity of HPD (Figure 3F). The above results not only indicate that HPP inhibits the methylation activity of HPD by competing with SAM for the active site but also indicate that the metabolic function and methylation function of HPD share the active site (Figure 3G).

### 2.6 HPD Exerts Methyltransferase Activity on Targeted mRNA through RRACH Motif

**Figure 4:**
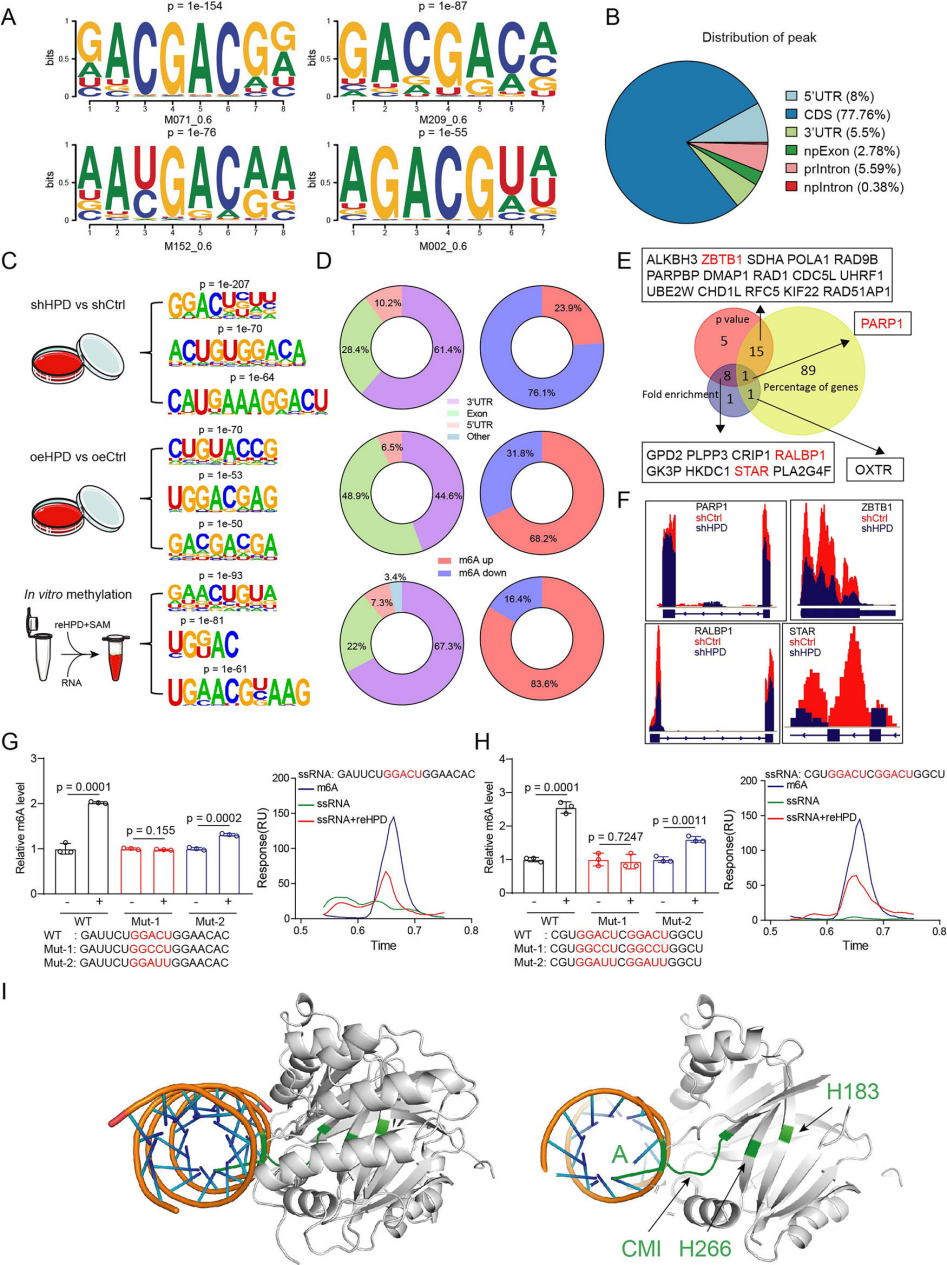
Identification of modification sites for HPD methyltransferases. A) The RNA binding motif of HPD was analyzed in eCLIP‐seq. B) The eCLIP‐seq was analyzed to found the region of mRNA bound by HPD. C,D) The MeRIP‐seq was performed in HCT116 cells and in vitro methylation assay samples. (shCtrl, *n* = 3; shHPD, *n* = 3; oeCtrl, *n* = 3; oeHPD, *n* = 3; Ctrl, *n* = 3; reHPD, *n* = 3). Flowchart for screening and identification of HPD modification sites. E) Bioinformatic analysis was performed to screen target genes subject to HPD‐regulated m^6^A modifications. F) The relative abundance of m^6^A peaks along PARP1, ZBTB1, RALBP1, and STAR mRNA in MeRIP‐seq. G,H) The in vitro methylation assay was employed to assess the methyltransferase activity of reHPD on single‐stranded RNA (ssRNA). The m^6^A modification was detected by colorimetric assay and LC/MS. I) Molecular docking analysis between protein HPD (PDB ID: 3ISQ) and RNA. Error bars in G and H, represent mean values ± SD, *p* values were determined by an unpaired two‐tailed Student's *t* test of *n* = 3 technical replicates.

To determine HPD and RNA binding and methylation sites, we performed eCLIP‐seq in HPD overexpressing cells. As a result, we found that HPD could not only bind to RNA, but also its binding region was consistent with the m^6^A modification motif (RRACH) (**Figure** **4**A). In addition, the binding peaks were distributed in the coding region (CDS), 3′UTR, and 5′UTR, and were mainly distributed in the CDS (Figure 4B). The RRACH motif has been identified as a conserved motif of the m^6^A modification site by MeRIP‐seq.^[^ ^23^, ^24^ ^]^ Then, we performed MeRIP‐seq in HPD‐knockdown cells, HPD‐overexpressed cells, and in vitro methylation samples to analyze the motif (Figure 4C). The results indicate that methylation modification increased in over 68% and 80% of the mRNA in the HPD‐overexpressed group and in vitro methylated group, while it decreased in over 75% of the mRNA in the HPD‐knockdown group, as compared to the control group (Figure 4D). Gene enrichment analysis found that m^6^A modifications on PARP1, ZBTB1, RALBP1, and STAR were regulated by HPD (Figure 4E,F). Consistent with the sequencing results, MeRIP‐qPCR results indicated that m^6^A modification on these genes was decreased by HPD knockdown (Figure S5A, Supporting Information). In our previous work, we found that the demethyltransferase FTO alters the m^6^A modification on PARP1 to regulate DNA damage.^[^ ^2^ ^]^ However, we did not find its m^6^A methyltransferase, because METTL3 does not alter its methylation level (Figure S5B, Supporting Information). Then, we performed in vitro methylation experiments using single‐stranded RNA (ssRNA) of PARP1 containing the m^6^A modification site, and the results showed that the m^6^A modification site of PARP1 is regulated by HPD (Figure 4G). The mutational assay reconfirmed the modification site, revealing that mutating A completely eliminated the methylation modification, while mutating C also significantly reduced the methylation modification on the RNA (Figure 4G). The synthetic single‐stranded RNA containing two typical GGACU also showed the same results (Figure 4H). The secondary structures of HPD and METTL3/METTL16 are similar. That is the α‐helix and β‐sheet are distributed alternately,^[^ ^25^ ^]^ and the key sites (H183, H266, and CMI) identified by us show a concentrated distribution (Figure S5C, Supporting Information). The molecular docking analysis between RNA (m^6^A‐modified region of PARP1) and HPD showed that H183, H266, CMI, and the modification site adenine (A) formed a special intermolecular route, which provides spatial structural convenience for the methylation process (Figure 4I). In short, the mRNA modification site of HPD is identical to that of METTL3 and also conforms to the RRACH motif.

### 2.7 HPD Regulates Ferroptosis by Altering SLC7A11/GPX4 mRNA Stability

**Figure 5:**
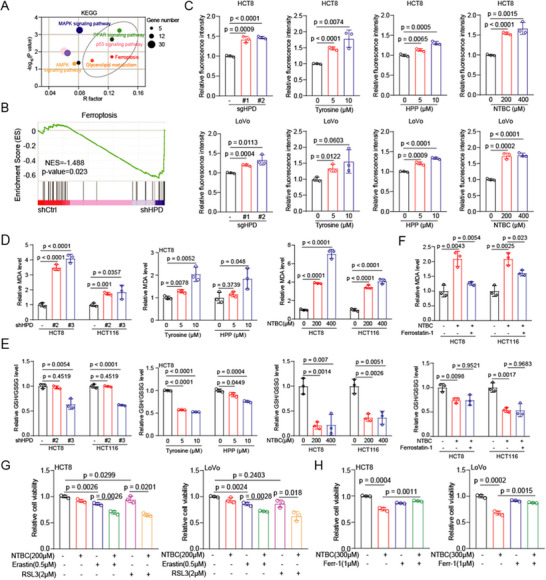
HPD regulates ferroptosis. A) The pathways involved in HPD were analyzed in RNA‐seq by KEGG. B) Ferroptosis was enrichment in HPD related pathway in RNA‐seq by GSEA. C) The lipid peroxidation level was detected in HPD knockout cells and cells treated with Tyrosine, HPP, and NTBC. D,E) The MDA level and GSH/GSSG ratio were detected in HPD knockdown and cells treated with Tyrosine, HPP, and NTBC. F) The MDA level and GSH/GSSG ratio were detected in NTBC treated cells plus with or without Ferrostatin‐1. G) The cell viability was determined in CRC cells treated with NTBC plus ferroptosis inducers (Erastin or RSL3). H) The cell viability was determined in CRC cells treated with NTBC plus ferroptosis antagonist (Ferrostatin‐1). Error bars in C, D, E, F, G, and H, represent mean values ± SD, *p* values were determined by unpaired two‐tailed Student's *t* test of *n* = 3 independent biological experiments.

To explore the biological role of HPD in CRC, we analyzed HPD‐related pathways and found that ferroptosis may be regulated by HPD (**Figure** **5**A,B). The lipid oxidation level is the key indicator of ferroptosis. We found that the lipid oxidation level was elevated in HPD knockout cells and Tyrosine/HPP/NTBC treated cells (Figure 5C). In addition, we also found a significant increase in the level of the lipid oxidation product MDA and a significant decrease in the GSH/GSSG ratio in HPD knockdown cells and Tyrosine/HPP treated cells (Figure 5D,E). Surprisingly, the MDA level, rather than the GSH/GSSG ratio, could be antagonized by ferroptosis inhibitors (Figure 5F). Similar to the MDA levels, the upregulation of 4‐HNE expression by NTBC was antagonized by ferroptosis inhibitors (Figure S6, Supporting Information). Lastly, we treated cells with a ferroptosis inducer or inhibitor, respectively, in combination with or without an HPD inhibitor (NTBC). We found that pharmacological inhibition of HPD alters the susceptibility of CRC cells to ferroptosis (Figure 5G,H), suggesting that HPD is involved in the regulation of ferroptosis.

**Figure 6:**
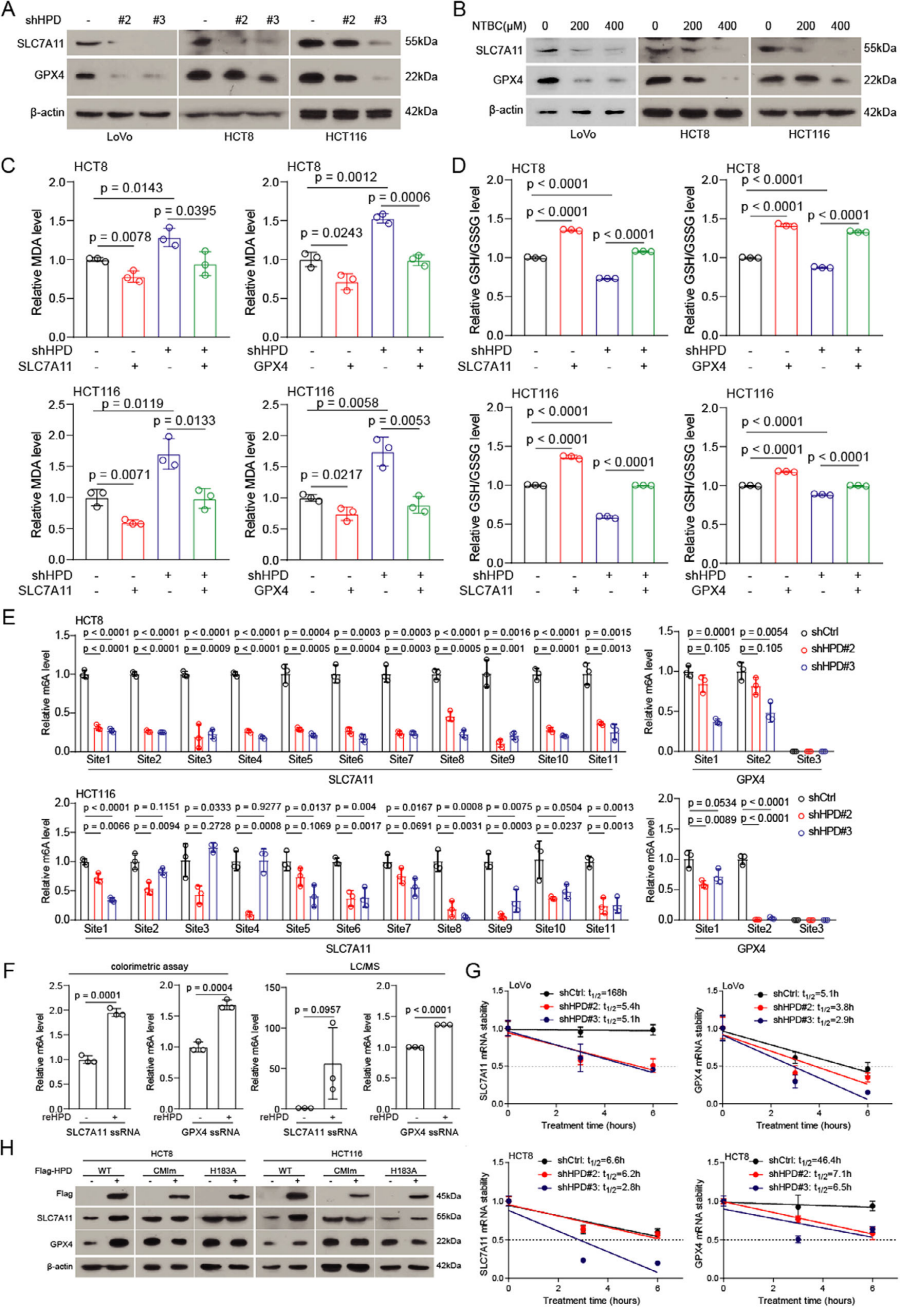
HPD exerts methyltransferase activity to regulate the expression of SLC7A11 and GPX4. A,B) The SLC7A11 and GPX4 proteins were detected in HPD knockdown cells and NTBC treated cells. C,D) The MDA level and GSH/GSSG ratio were detected in HPD knockdown cells with or without re‐overexpressing SLC7A11/GPX4. E) The m^6^A modification was detected in HPD knockdown and control groups by MeRIP‐qPCR. F) The in vitro methylation assay was employed to assess the methyltransferase activity of reHPD and SLC7A11/GPX4 ssRNA (*n* = 3). The m^6^A modification was detected by colorimetric assay and LC/MS. G) The mRNA stability of target genes was detected in the HPD knockdown and control groups. H) The expression of target proteins was detected in HPD (wild‐type and inactivating mutant) overexpressing cells. Error bars in C, D, E, F, and G, represent mean values ± SD, *p* values were determined by unpaired two‐tailed Student's *t* test of *n* = 3 independent biological experiments.

To explore the molecular mechanism by which HPD regulates ferroptosis, we examined the expression of ferroptosis‐related factors using quantitative PCR and immunoblotting assays. We found that HPD dysfunction significantly down‐regulated the expression of SLC7A11 and GPX4 (**Figure** **6**A,B; Figure S7A–D, Supporting Information). Importantly, the MDA level and GSH/GSSG ratio were restored not only by the overexpression of SLC7A11, but also by the overexpression of GPX4 (Figure 6C,D). The presence of m^6^A modifications on three target genes could be detected in tumor cells, and methylation modification was upregulated in HPD overexpressing cells (Figure S7E,F, Supporting Information). Based on previous studies,^[^ ^26^ ^]^ we found that the knockdown of HPD significantly reduced m^6^A modification in specific regions of SLC7A11 and GPX4 (Figure 6E). We also synthesized single‐stranded RNA of SLC7A11 and GPX4. In vitro results showed that HPD could up‐regulate the methylation level of single‐stranded RNA (Figure 6F). The m^6^A modification regulates mRNA splicing, translocation, stability, and translation efficiency. Here, we found that HPD reduces the stability of target gene mRNAs (Figure 6G; Figure S7G, Supporting Information). We also found that wild‐type HPD, rather than methyltransferase‐inactivated mutant HPD, up‐regulates the expression of target proteins in HPD overexpressing cells (Figure 6H). This suggests that the regulation of HPD on target gene expression is dependent on methyltransferase activity. In conclusion, we found that HPD is dependent on methyltransferase activity to regulate the expression of target genes (SLC7A11 and GPX4), thereby modulating the onset of ferroptosis.

### 2.8 HPD Depends on Methyltransferase Activity to Promote CRC Growth

**Figure 7:**
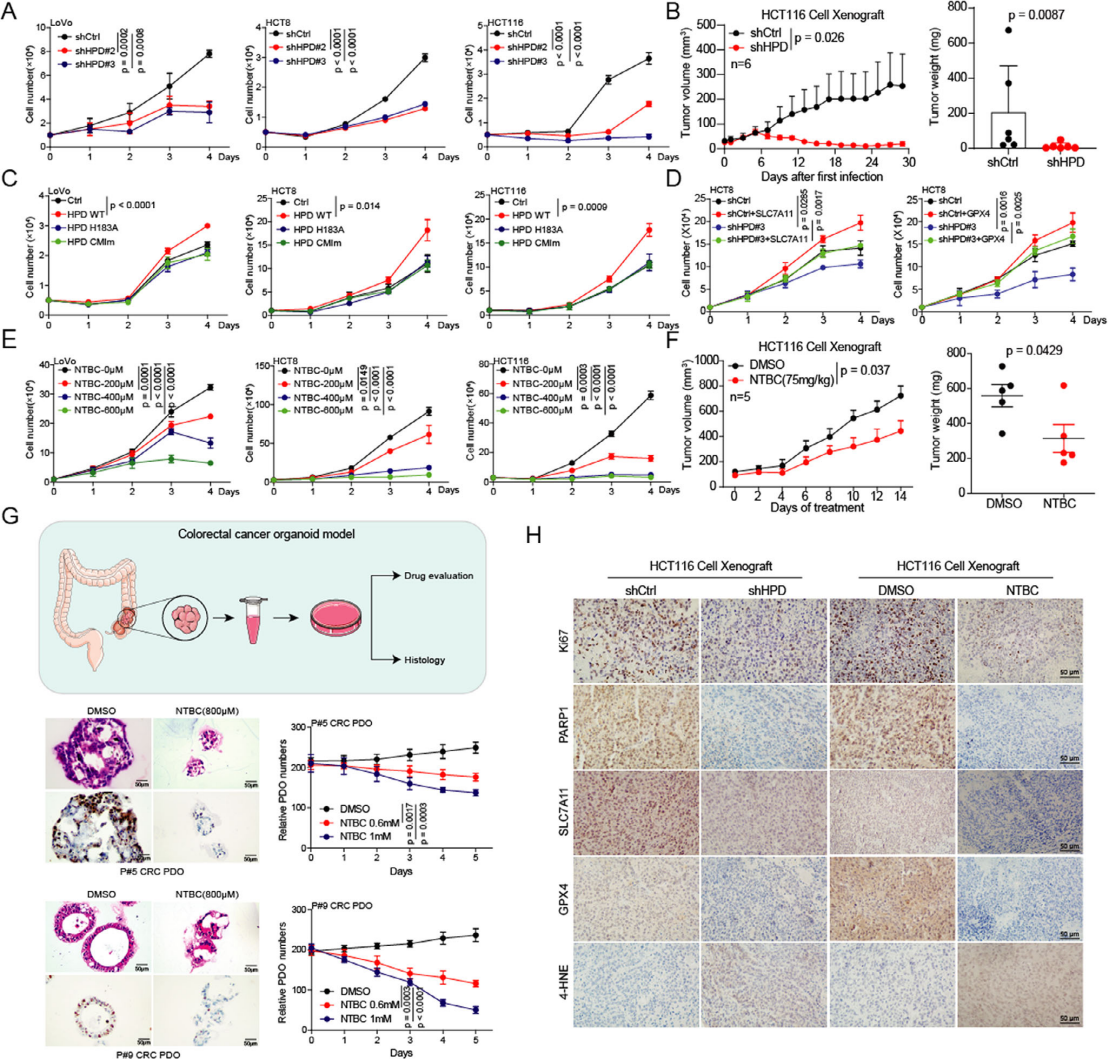
HPD exerts methyltransferase activity to promote colorectal cancer progression. A) Cell proliferation was determined in the HPD knockdown and control groups. B) Tumor growth rates and tumor weights were recorded in the HCT116‐CDX model (*n* = 6). C) Cell proliferation was determined in overexpressing HPD (wild‐type and inactivating mutant) cells. D) Cell proliferation was determined in HPD knockdown cells with or without re‐overexpressing SLC7A11/GPX4. E) Cell proliferation was determined in NTBC treated cells. F) Tumor growth rates and tumor weights were recorded in the HCT116‐CDX model (*n* = 5). G) Organoid growth was determined in CRC cells treated with NTBC. HE staining and Ki67 immunostaining were performed in the NTBC‐treated CRC organoid model. H) The target proteins and ferroptosis markers were detected by immunohistochemistry in the HCT116‐CDX model. Error bars in A, C, D, E, F, and G, represent mean values ± SD, *p* values were determined by unpaired two‐tailed Student's *t* test of *n* = 3 independent biological experiments.

The m^6^A modification plays an important role in tumor progression.^[^ ^27^, ^28^, ^29^ ^]^ To determine the role of HPD in colorectal cancer, we knocked down HPD and found that the cell proliferation and tumor growth of CRC cells were significantly inhibited (**Figure** **7**A,B; Figure S8A,B, Supporting Information). The same results were also observed in the CDX and PDX models with the HPD knockdown (Figure S8C, Supporting Information). To determine the role of HPD's methyltransferase activity in cell proliferation, we generated inactive mutants. The results showed that overexpression of the methyltransferase inactivating mutant did not promote the cell proliferation of CRC cells compared with the wild type (Figure 7C), which indicates that HPD promotes cell proliferation depending on methyltransferase activity. In addition, cell proliferation was restored not only by overexpression of SLC7A11 but also by overexpression of GPX4 (Figure 7D; Figure S8D,E, Supporting Information). These results indicate that HPD, which regulates SLC7A11/GPX4 expression and tumor cell growth, acts as a methyltransferase.

Targeted small molecules are an important source and raw material library for drug research and development. We treated CRC cells with NTBC and metabolites (Tyrosine and HPP), and found that NTBC and metabolites (Tyrosine and HPP) inhibited CRC cell proliferation (Figure 7E; Figure S8F, Supporting Information). Moreover, NTBC also exhibits tumor suppressive ability in the CDX model (Figure 7F; Figure S8G, Supporting Information). Organoids have become an important tool for anti‐tumor research and drug development.^[^ ^30^ ^]^ We established two CRC patient‐derived organoid models of colorectal cancer, and found that NTBC significantly inhibits CRC growth (Figure 7G; Figure S8H, Supporting Information). To validate the correlation of HPD with DNA damage and ferroptosis, we collected tumor tissues from animal models and applied immunoblotting and immunohistochemistry to examine the effect of HPD on DNA damage and ferroptosis markers. First, our results revealed a significant reduction of m^6^A modification, a significant down‐regulation of the expression of target proteins, and a significant up‐regulation of biomarkers of DNA damage and ferroptosis in HPD knockdown or NTBC‐treated groups (Figure 7H; Figure S9, Supporting Information). In conclusion, we found that HPD promotes tumor progression in CRC, which is dependent on its methyltransferase activity.

## 3 Discussion

In this study, we report that the tyrosine metabolism enzyme HPD enters the nucleus and acts as an m^6^A methyltransferase. Specifically, HPD exerts its m^6^A methyltransferase activity depending on the catalytic mode sequence (RPTLF, CMI) and SAM binding sites (H183 and H266). Our finding adds to the understanding of this phenomenon: the m^6^A modification was added by the known m^6^A methyltransferase METTL3, which recruits METTL14 and WTAP, to form a complex. However, HPD does not require the assistance of other proteins and installs m^6^A modification on mRNA. Here, we demonstrate that HPD methylates SLC7A11/GPX4 through a moonlighting function, which suppresses CRC ferroptosis and promotes tumor growth. Thus, targeting the m^6^A methyltransferase activity of HPD leads to inducing CRC ferroptosis and blocking tumor growth.

HPD was considered a tyrosine metabolizing enzyme whose metabolic end products would enter the gluconeogenic and ketogenic pathways. The process of tyrosine metabolism is thought to occur in the cytoplasm,^[^ ^31^, ^32^ ^]^ whereas HPD catalyzes methylation modifications that occur in the nucleus. Therefore, we believe that the two enzymatic activities of HPD are distinguished based on cellular localization. Structurally, HPD can be broadly divided into N‐terminal and C‐terminal. Its N‐terminal has been found to mainly maintain the stability of the structural domain,^[^ ^33^ ^]^ while the C‐terminal serves as an active pocket region for binding physiological substrates. Both the catalytic mode sequence (RPTLF) and the SAM binding sites we identified are located at the C‐terminus. More importantly, the tyrosine metabolizing enzyme activity was severely disrupted when we mutated the CMI motif and the SAM binding sites. This may indicate that tyrosine metabolizing enzymes and methyltransferases share the same activity pocket. Although the upstream metabolites of HPD, tyrosine and HPP, can reduce its methyltransferase activity and induce ferroptosis, we still cannot determine whether it is accomplished by inhibiting the enzymatic activity of HPD or enhancing its metabolic process, which requires further study. HPD is a multifunctional enzyme that plays a crucial regulatory role in various biological processes, exhibiting three core biochemical activities: tyrosine‐metabolizing, RNA‐binding, and methyltransferase activities. The observed functional overlap among these activities may stem from a shared active pocket. Therefore, investigating a particular function of HPD requires careful consideration and control of potential interference from its other catalytic roles.

To explore the role of HPD in CRC, we conducted KEGG analysis and found that ferroptosis was enriched in the HPD knockdown group. Inducing ferroptosis in tumor cells has emerged as a potential strategy for clinical treatment, with SLC7A11 and GPX4 being crucial drug targets.^[^ ^34^, ^35^ ^]^ Here, we demonstrate that HPD relies on methyltransferase activity to regulate the stability of SLC7A11 and GPX4 mRNA, thereby influencing their expression. Genetic perturbation or pharmacological targeting of HPD can promote ferroptosis and impede CRC tumor progression. However, we observed that the METTL3/14 complex and HPD exert opposing effects on the expression levels of SLC7A11 and GPX4,^[^ ^26^ ^]^ two key regulators involved in cellular antioxidant defense mechanisms. This pivotal discovery serves to underscore the profound complexity inherent in m^6^A‐mediated epigenetic regulation. Rather than functioning as a straightforward molecular on/off switch, the evidence indicates that m^6^A modification operates as a highly dynamic and integrated regulatory network. This system relies on the coordinated and context‐dependent interplay among writer complexes, eraser enzymes, and reader proteins, each contributing to fine‐tuned control over gene expression and subsequent functional outcomes in response to cellular signals and environmental cues.

## 4 Conclusion

Collectively, we identify HPD as a novel m^6^A methyltransferase, and explore the underlying mechanisms by which it exerts m^6^A methyltransferase activity. Meanwhile, these results provide insights into not only the metabolic but also the non‐metabolic functions of HPD in regulating tumor progression. However, there are several outstanding issues that remain. First, we did not explore how HPD coordinates tyrosine metabolizing enzyme activity and methyltransferase activity under physiological conditions. Second, how HPD is imported and localized to the nucleus has not yet been elucidated. Thus, further studies are needed to elucidate the molecular mechanisms by which HPD shuttles across the nuclear membrane.

## 5 Experimental Section

### Reagents and Biological Resources

The Reagents and Biological Resources are listed in the key resources table of Supporting Information.

### Cell Culture

The colorectal cancer cell lines LoVo, HCT8, and HCT116 were cultured in RPMI‐1640 medium supplemented with 10% FBS (ExCell, Shanghai, China) and penicillin/streptomycin. HEK293T cells were cultured in DMEM supplemented with 10% FBS and penicillin/streptomycin. All cells were cultured at 37 °C and 5% CO_2_.

### Cell Immunofluorescence

The cells were cultured on coverslips for 24 h before experimental treatment. Samples were first fixed with 4% paraformaldehyde for 15 min, rinsed three times with PBS, permeabilized with 0.1% Triton X‐100 in PBS for 20 min, and then blocked with 2% BSA in PBS for 60 min. For RNase A‐sensitivity analysis, cells were treated with RNase A (Beyotime, ST578) at 37 °C for 20 min after being permeabilized. The samples were incubated with indicated the indicated primary antibodies diluted in 2% BSA in PBS overnight at 4 °C and rinsed three times with PBS before a 30 min incubation with secondary antibodies conjugated to Alexa Fluor‐555/488. After being washed three times with PBS, the coverslips were mounted using DAPI Fluoromount‐G. Fluorescent micrographs were obtained using laser scanning confocal microscopy (Leica, Wetzlar, Germany).

### RNA Sequencing, MeRIP Sequencing and eCLIP Sequencing

After the HPD knockdown cells and control cells were subjected to immunoblotting to verify the knockdown, 5 × 10^6^ cell precipitates were collected and resuspended with 1 mL of TRIzol reagent. Then quickly frozen and underwent MeRIP‐seq and RNA‐seq. For in vitro methylation assay samples, the samples were purified and sequenced. The coverage of m^6^A peaks reads was shown by IGV software. After immunoblotting of HPD‐overexpressing cells and control cells to confirm overexpression, cell pellets were collected and subjected to eCLIP sequencing (Wuhan Kangce Technology Co., Ltd.). The data generated in this study are publicly available in the sequence read archive (SRA) database (accession numbers: PRJNA1124845, PRJNA1126087, PRJNA1126437, and PRJNA1205343).

### Dot Blot

A dot blot assay was conducted to determine the global m^6^A abundance of total RNA or mRNA. The mRNA was enriched using the PolyATract mRNA isolation System IV (Promega, Z5310) following the manufacturer's instructions. In brief, total RNA or mRNA was mixed with 1 × SSC (saline sodium citrate) buffer and denatured at 65 °C for 5 min. Then, the RNA samples were loaded on the Amersham Hybond─N + membrane (GE Healthcare, RPN119B), and cross‐linked to the membrane by UV. The membrane was stained with methylene blue as a control. Then, the membrane was blocked with 5% skim milk and incubated with the m^6^A antibody overnight at 4 °C. After being rinsed with 1×PBST, the membrane was incubated with the secondary antibody for 1 h. The signal was detected using the Immobilon Western HRP Kit (Millipore, USA).

### m^6^A Quantification by the m^6^A RNA Methylation Quantification Kit (Colorimetric)

The m^6^A modification was assayed using an m^6^A assay kit according to the manufacturer's instructions (Epigentek, P‐9005). Specifically, 200 ng of total RNA was added to a 96‐well plate containing binding solution, gently mixed and incubated at 37 °C for 90 min. After rinsing three times with wash buffer, the diluted captured antibody was added and incubated for 60 min at room temperature. After discarding the supernatant, the plated was rinsed three times with wash buffer, the diluted detection antibody was added and incubated at room temperature for 30 min. After discarding the supernatant again, the plated was rinsed four times with wash buffer, the diluted enhancer solution was added and it was incubated at room temperature for 30 min. After discarding the supernatant once more, the plated was rinsed five times with wash buffer, the diluted developer solution was added, and it was incubated at room temperature for 1–10 min. The stopping solution was added to terminate the reaction and read on a microplate reader at 450 nm within 2–15 min.

### LC/MS

Total RNA was extracted from the collected cells and subjected to mRNA purification using a PolyATtract mRNA Isolation System (Promega, Z5310). The mRNA concentration was measured by ultraviolet absorbance at 260 nm. mRNA (200 ng) was first denatured by heating at 95 °C for 5 min, chilled on ice for 2 min, and added with 1/10 volume of P1 nuclease buffer and 50 units of P1 nuclease (New England Biolabs, M0660S). The mixture (20 µL) was incubated at 37 °C for 3 h. The solution was subsequently added to 1/10 volume of alkaline phosphatase buffer and 5 units of alkaline phosphatase (Beyotime, D7027). The mixture was continuously incubated at 37 °C for an additional 4 h, followed by extraction with an equal volume of chloroform twice. The resulting aqueous layer was collected, lyophilized to dryness, and reconstituted in 100 µL of water. The sample was diluted to 1 mL and then filtered (0.22 µm pore size, 4 mm diameter, Millipore). The solution (10 µL) was injected into the LC/MS device. The nucleosides were separated by reverse‐phase ultra‐performance liquid chromatography on a C18 column with online mass spectrometry detection using Waters XEVO TQ‐STM (Waters, USA) triple quadrupole mass spectrometer in positive electrospray ionization mode. All nucleosides were quantified using retention time and ion mass transitions of 268.0 to 136.0 (A) and 282.1 to 150.0 (m^6^A). For single‐stranded RNA synthesized in vitro, the ssRNA content in the in vitro methylation assay was 1 µg.

### Western Blot

The cells were harvested and lysed using the RIPA lysis buffer at 4 °C. The cell lysate was centrifuged and the protein concentration was quantified using an ultraviolet spectrophotometer. An equal amount of cell lysate from each sample was loaded onto the SDS‐PAGE. The proteins were transferred onto PVDF membranes (Millipore, USA), blocked with 5% skim milk and incubated with primary antibodies. HRP conjugated goat anti‐mouse or anti‐rabbit IgG was used as a secondary antibody. The signal was detected using Immobilon Western HRP Kit (Millipore, USA).

### Cytoplasmic and Nuclear Protein Separation Assay

The cells were partitioned into cytoplasmic and nuclear fractions using a nuclear and cytoplasmic protein extraction kit (Beyotime, P0027) according to the manufacturer's instructions.

### Co‐Immunoprecipitation

The Flag‐conjugated beads or agarose pre‐conjugated with the appropriate antibody for 2 h were added to the supernatant and incubated overnight at 4 °C. Immunoprecipitates obtained by centrifugation were washed three times with pre‐chilled PBS and subjected to western blotting using specific antibodies.

### Quantitative Real‐Time PCR

Total RNA isolated using the TRIzol reagent was subjected to reverse transcription and RT‐qPCR. Gene expression was calculated using the comparative 2^−ΔΔCT^ method with the actin for normalization. All primers used in this study are listed in the key resources table of Supporting Information.

### Protein Expression and Purification

For bacterial expression of HPD and METTL3 proteins (including wild type and mutant), the His6 × tag containing pET‐M3C vector was transformed into BL21 competent cells. Bacterial cultures were grown at 37 °C for 4 h. Then, isopropyl‐β‐D‐1‐thiogalactopyranoside (IPTG) was added to a final concentration of 1 mm for 20 h of induction at 18 °C. Bacterial cells were spun down at 8000 rpm for 10 min, resuspended and lysed in 20 mm Tris‐HCl supplemented with 500 mm NaCl. After brief sonication, the lysates were centrifuged 12 000 rpm for 30 min at 4 °C, and supernatants were collected. The supernatants were passed through a Ni‐column (Qiagen, 30250) and washed sequentially with the following buffers: 10 mm imidazole, 30 mm imidazole, 50 mm imidazole. The His6 ×‐labeled target proteins were eluted in 250 mm imidazole, and 20 µL of the eluate was analyzed by SDS‐PAGE. Fractions containing purified target proteins were combined and concentrated using microcon‐10 filter (Millipore, MRCPRT010) and stored at −80 °C.

For eukaryotic cell expression of HPD and METTL3 proteins (including wild type and mutant), after the Flag‐tagged fusion protein plasmid was transfected into the cells, the cells were collected after 48 h of culture. Similar to immunoprecipitation, and the target proteins were enriched using Flag‐beads at 4 °C overnight. The next day, the target proteins were competitively eluted using 3 × Flag peptide, followed by protein quantification. The proteins were then stored for follow – up experiments at −80 °C.

### In Vitro Methylation Assay

In vitro methylation reactions were performed in 50 µL reaction system with METTL3 or HPD. The cells were transfected with vectors expressing Flag‐METTL3 and Flag‐HPD. After 48 h, cell lysates were prepared and subjected to immunoprecipitation assays to enrich Flag‐METTL3 and Flag‐HPD proteins. The recombinant proteins (reMETTL3 and reHPD) were expressed and enriched in *E. coli*. The reaction mixture containing METTL3 or HPD (30 nm), total RNA (100 µg) or single‐stranded RNA (1 µg), and a reaction buffer including 0.8 mm S‐adenosyl‐L‐methionine (SAM), 80 mm KCl, 1.5 mm MgCl_2_, 0.2 U mL^−1^ RNasin, 10 mM DTT, 4% glycerol, and 15 mm HEPES (pH 7.9). The reactions were incubated at 30 °C for 2 h and stopped by the addition of SSC buffer for dot blot.

### Tyrosine Metabolic Enzyme Activity

To detect the tyrosine metabolic enzyme activity of HPD, the expression plasmids of HPD (wild type and mutant) were transformed into *E.coli* (DE3) and cultured overnight. A single colony was picked and inoculated into 5 mL of LB medium, then incubated overnight at 37 °C, 220 rpm. On the second day, 10 µL of bacterial suspension was transferred to a new 5 mL LB medium with tyrosine, and incubated at 37 °C, 220 rpm for 3.5 h. IPTG with a final concentration of 1 mg mL^−1^ was added to the medium to induce the expression of HPD protein. After 8 h of culture at 16 °C with shaking at 160 rpm, the culture was continued overnight at 37 °C, 220 rpm. Finally, the supernatant of the bacterial suspension was collected, and the absorbance value was measured at a wavelength of 405 nm.

### Thermal Shift Assay

The recombinant protein was diluted in 1 × PBS and divided into two equal aliquots. SAM (20 µm) or a control was added to the supernatant, and the mixture was incubated at 25 °C for 30 min. After denaturing at various temperatures for 30 min, the samples were centrifuged, and the supernatants were analyzed by western blot.

### MeRIP‐qPCR

The total RNA was isolated and an equal amount of RNA was incubated with m^6^A antibody or control rabbit IgG mixed Protein A/G Beads (Santa Cruz, CA) in 500 µL buffer containing 40 U RNase inhibitors overnight at 4 °C. RNA with m^6^A modification was immunoprecipitated by m^6^A antibody‐conjugated beads, washed three times, and then incubated with proteinase K digestion buffer. RNA was finally purified by TRIzol/chloroform extraction and analyzed by RT‐qPCR. For identification of specific m^6^A sites, 10× fragmentation buffer was used (mix 800 µL of molecular biology‐grade, RNase‐free water with 100 µL (1 m stock) of Tris‐HCl (pH 7.0) and 100 µL (1 M stock) of ZnCl_2_. Freshly prepare the buffer. The final concentrations in the buffer concentrate are 100 mm Tris‐HCl and 100 mm ZnCl_2_) to fragment the RNA prior to incubation. The qPCR primers need to be designed on both sides of the predicted m^6^A site and are listed in the key resources table of Supporting Information.

### MDA Level Assay

Malondialdehyde (MDA) was a terminal product of lipid peroxidation. For the MDA assay, cell proteins were prepared according to the description in the Lipid Peroxidation MDA assay kit (Beyotime, S0131). The MDA level was detected using a microplate reader at 532 nm.

### GSH/GSSG Ratio Assay

The intracellular GSH/GSSG ratio was measured using a GSH/GSSG assay kit (Beyotime, S0053) according to the manufacturer's instructions. The collected cells were added protein removal reagent, and then rapidly freeze‐thawed twice using liquid nitrogen and a 37 °C water bath, after which the samples were placed on ice for 5 min and centrifuged for 10 min at 4 °C. Take the supernatant for subsequent Total Glutathione and GSSG assays. Total Glutathione and GSSG were measured in the supernatant by reading the fluorescence at 405 nm. Finally, according to the measured total glutathione content and GSSG content, the GSH content can be calculated (GSH = Total Glutathione‐GSSG × 2).

### Lipid Peroxidation Measurement

Cells were treated as indicated, and then 2 µM C11‐BODIPY (Beyotime, S0043) was added, and the cells were incubated for 30 min. Excess C11‐BODIPY was removed by washing the cells twice with PBS. The lipid ROS level was detected using a microplate reader at specific wavelengths. The maximum excitation and emission wavelengths of the reduced product of BODIPY 581/591 C11 were 581/591 nm, which shifted to ≈488/510 nm after oxidation by lipid hydroperoxide.

### WST‐1 Assay

Cell viability was measured using a WST‐1 Cell Proliferation and Cytotoxicity Assay Kit (Beyotime, C0036L). Cells were seeded in 96‐well plates and treated as indicated. The WST‐1 reagent was added, and cells were incubated at 37 °C for 1 h. The absorbance was detected by a microplate reader at 450 nm.

### Immunohistochemistry

Immunohistochemistry was performed on paraffin‐embedded sections. Tissue sections were dewaxed and rehydrated following a standard protocol. Antigen retrieval was carried out by boiling samples in citrate buffer for 15 min. Endogenous peroxidase activity was inhibited by using 3% hydrogen peroxidase. Sections were blocked in 3% BSA in PBS and incubated in primary antibody overnight at 4 °C. Sections were rinsed in PBS and developed using DAB. The sections were counterstained with hematoxylin. Quantification of the staining was performed using Image J software.

### Animal Models

For the CDX model, nude mice (female, 4–6‐week‐old) were subcutaneously injected with 5 × 10^6^ HCT116 cells on both flank. For the PDX model, the fresh CRC tumor tissues used to establish the PDX mice model were provided by Tianjin Union Medical Center (Tianjin, China). The patient tumor tissues were divided into small pieces and then inoculated on both flank of nude mice. After the tumor grew to a suitable size, the tumor was divided into small pieces and then subcutaneously inoculated on both flank of other mice. For the knockdown HPD mice model, two weeks after inoculation, the shHPD#3 lenti‐virus injected into the tumor for three consecutive days. For NTBC treated mice model, intraperitoneal injection of NTBC was started one week after inoculation.

This study was carried out in accordance with the recommendations of Requirements of the Ethical Review System of Biomedical Research Involving Human by National Health and Family Planning Commission of China, Nankai University and Tianjin Union Medical Center Ethics Committee with written informed consent from all subjects (Ethical Approval No. NKUIRB2024055). All subjects gave written informed consent in accordance with the Declaration of Helsinki. Approval of use of mice and designed experiments was given by the Laboratory Animal Ethics Committee Nankai University (Ethical Approval No. 2024‐SYDWLL‐000094).

### Establishment of Patient‐Derived Organoid (PDO)

Primary tumor tissues obtained during surgery were cleaned with a washing solution (Precedo, Hefei, China) and cut into small pieces of 1–3 mm^3^. Then, a digestive enzyme (Precedo, Hefei, China) was used to digest the tissue at 37 °C until there were 3–10 cell clumps. The digested cell clumps were collected and then re‐suspended using Intestine Carcinoma Organoid Medium (Precedo, Hefei, China). Reduced growth factor basement membrane matrix Type 2 (BME, R&D Systems) was added and mixed to a final volume of 70%. The 24‐well plates were placed in a cell incubator at 37 °C to solidified the BME completely. Each well was added with 500 µL complete medium and placed in a cell incubator with 5% CO_2_ at 37 °C for further culture.

### Statistical Analysis

Data were analyzed using GraphPad Prism and were presented as the mean ± SD as indicated. A Two‐tailed Student's *t*‐test was used to compare means between groups as indicated. *p* <0.05 was considered as a statistically significant difference.

## Conflict of Interest

The authors declare no conflict of interest.

## Author Contributions

J.W., X.D., and H.L. contributed equally to this work. J.Y.W. was responsible for the drafting of the manuscript. J.Y.W., X.T.D., H.L.L., S.W.H., H.K.C., H.R.S., M.M.S., H.F.Z., K.M.N., F.X., Y.Y.Q., Q.L.G., C.X.Y., Q.J.Z., and J.S.G. performed acquisition of data, analysis and interpretation of data, and statistical analysis. J.Y.W., C.Z.Z., S.Z., and C.L.S. handled funding acquisition. S.Z. and C.L.S. were responsible for the study concept, design, and study supervision. C.L.S. specifically handled the Writing‐reviewing and editing. All authors have read and approved the final manuscript.

## Supporting information



Supporting Information

## Data Availability

The data that support the findings of this study are available on request from the corresponding author. The data are not publicly available due to privacy or ethical restrictions.
