# Supplementary material for: HPD is an m6A Methyltransferase that Protects Colorectal Cancer Cells from Ferroptotic Cell Death by m6A Methylating SLC7A11/GPX4
Source: Adv Sci (Weinh). 2025 Nov 29;13(9):e08541. doi: 10.1002/advs.202508541 (PMC12903970; doi:10.1002/advs.202508541)

Supporting Information

HPD is an m^6^A Methyltransferase that Protects Colorectal Cancer Cells from Ferroptotic Cell Death by m^6^A Methylating SLC7A11/GPX4

Jiyan Wang^#^, Xintong Dai^#^, Huanle Liu^#^, Saiwei Hua, Hongkai Chang, Huanran Sun, Mingming Sun, Huifang Zhao, Kemin Ni, Fei Xie, Yaya Qiao, Qingle Gao, Chenxi Yu, Qijun Zhang, Jianshuang Guo, Chunze Zhang, Shuai Zhang, Changliang Shan*

J. Wang^#^, X. Dai^#^, H. Liu^#^ contributed equally to this work.

**Supporting Experimental Section/Methods**

*Reagents and Biological Resources*

**KEY RESOURCES TABLE**

| REAGENT or RESOURCE | SOURCE | IDENTIFIER |
| --- | --- | --- |
| Antibodies | | |
| HPD Rabbit PolyAb | proteintech | Cat# 17004-1-AP; RRID:AB_2264310 |
| HPD Rabbit McAb | proteintech | Cat# 67552-1-Ig; RRID:AB_2882766 |
| PARP1 Mouse McAb | proteintech | Cat# 66520-1-Ig; RRID:AB_2881883 |
| PARP1 Rabbit PolyAb | proteintech | Cat# 13371-1-AP; RRID:AB_2160459 |
| Alpha-Tubulin Rabbit PolyAb | proteintech | Cat# 11224-1-AP; RRID:AB_221020 |
| Bata Actin Mouse McAb | proteintech | Cat# 66009-1-Ig; RRID:AB_2687938 |
| Flag Rabbit PolyAb | proteintech | Cat# 20543-1-AP; RRID:AB_11232216 |
| SLC7A11 Rabbit PolyAb | proteintech | Cat# 26864-1-AP; RRID:AB_2880661 |
| GPX4 Rabbit PolyAb | proteintech | Cat# 30388-1-AP; RRID:AB_3086304 |
| METTL3 Rabbit PolyAb | proteintech | Cat# 15073-1-AP; RRID:AB_2142033 |
| METTL14 Rabbit PolyAb | proteintech | Cat# 26158-1-AP; RRID:AB_2800447 |
| ALKBH5 Rabbit PolyAb | proteintech | Cat# 16837-1-AP; RRID:AB_2242665 |
| FTO Rabbit PolyAb | proteintech | Cat# 27226-1-AP; RRID:AB_2880809 |
| Ki67 Rabbit PolyAb | Cell signaling technology | Cat# 9027; RRID:AB_2636984 |
| γH2A.X | Millipore | Cat# 05-636; RRID: AB_309864 |
| N6-methyladenosine | Abcam | Cat# ab190886 |
| N6-methyladenosine | Abcam | Cat# ab151230; RRID:AB_2753144 |
| SC35 | Abcam | Cat# ab11826; RRID:AB_298608 |
| 4-HNE | Abcam | Cat# ab46545; RRID:AB_722490 |
| Bacterial and virus strains | | |
| Trans10 Chemically Competent Cell | [TransGen Biotech](http://www.baidu.com/link?url=2lik0ADTVVBIG_Pn4V7hg4i7OGiZ9dMUqQ2Jzk3lPlFwz-SOqDvqkcZ8TmHxXHuhSZDDcbrhBCMMD-0RfRDnCq) | Cat# CD101-01 |
| Trans BL21 (DE3) Chemically Competent | [TransGen Biotech](http://www.baidu.com/link?url=2lik0ADTVVBIG_Pn4V7hg4i7OGiZ9dMUqQ2Jzk3lPlFwz-SOqDvqkcZ8TmHxXHuhSZDDcbrhBCMMD-0RfRDnCq) | Cat# CD601-03 |
| Biological samples | | |
| Human colorectal cancer samples for PDX | Department of Colorectal Surgery, Tianjin Union Medical Center | N/A |
| Human colorectal cancer samples for PDO | Department of Colorectal Surgery, Tianjin Union Medical Center | N/A |
| Chemicals, peptides, and recombinant proteins | | |
| Nitisinone | Sigma | Cat# PHR1731 |
| SAM | Sigma | Cat# A2408 |
| Polybrene | Sigma | Cat# H9268 |
| 4-Hydroxyphenylpyruvic acid | MedChemExpress | Cat# HY-W010040 |
| RSL3 | MedChemExpress | Cat# HY-100218A |
| Ferrostatin-1 | MedChemExpress | Cat# HY-100579 |
| 5-Fluorouracil | MedChemExpress | Cat# HY-90006 |
| Cisplatin | MedChemExpress | Cat# HY-17394 |
| Olaparib | MedChemExpress | Cat# HY-10162 |
| Adenosine | MedChemExpress | Cat# HY-B0228 |
| N6-Methyladenosine | MedChemExpress | Cat# HY-N0086 |
| Actinomycin D | MedChemExpress | Cat# HY-17559 |
| Erastin | TargetMol | Cat# T1765 |
| Protein A/G sepharose | GE Healthcare Life Sciences | Cat# 17-0618-01 |
| Puromycin | InvivoGen | Cat# Ant-pr-1 |
| Polyethylenimine (PEI) | Polysciences | Cat# 23966 |
| Normal goat serum (NGS) | ZSGB-BIO | Cat# ZLI-9021 |
| DAB | MXB Biotechnologies | Cat# DAB-0031/1031 |
| Immobilon Western Chemiluminescent HRP Substrate | Millipore | Cat# WBKLS0500 |
| TRIzol | Thermo Fisher Scientific | Cat# 15596018 |
| Trypsin 0.5% EDTA | Thermo Fisher Scientific | Cat# 25200-072 |
| DMEM Medium | Thermo Fisher Scientific | Cat# C11995500BT |
| RPMI 1640 Medium | Thermo Fisher Scientific | Cat# C11875500BT |
| Critical commercial assays | | |
| PrimeScript RT reagent Kit with gDNA Eraser | TaKaRa | Cat# RR047A |
| TB Green® Premix Ex TaqTM Ⅱ (Tli RNaseH plus) | TaKaRa | Cat# RR820A |
| TransStart FastPfu DNA Polymerase | [TransGen Biotech](http://www.baidu.com/link?url=2lik0ADTVVBIG_Pn4V7hg4i7OGiZ9dMUqQ2Jzk3lPlFwz-SOqDvqkcZ8TmHxXHuhSZDDcbrhBCMMD-0RfRDnCq) | Cat# AP221-12 |
| Fast mutagenesis system | [TransGen Biotech](http://www.baidu.com/link?url=2lik0ADTVVBIG_Pn4V7hg4i7OGiZ9dMUqQ2Jzk3lPlFwz-SOqDvqkcZ8TmHxXHuhSZDDcbrhBCMMD-0RfRDnCq) | Cat# FM111-01 |
| Nuclear and Cytoplasmic Protein Extraction Kit | Beyotime | Cat# P0028 |
| WST-1 Cell Proliferation and Cytotoxicity Assay Kit | Beyotime | Cat# C0036L |
| Lipid Peroxidation Assay Kit with BODIPY 581/591 C11 | Beyotime | Cat# S0043S |
| GSH and GSSG Assay Kit | Beyotime | Cat# S0053 |
| Lipid Peroxidation MDA Assay Kit | Beyotime | Cat# S0131M |
| m6A RNA Methylation Quantification Kit | Epigentek | Cat# P-9005 |
| Deposited data | | |
| RNA-seq (Raw sequencing data) | This paper | NCBI SRA: PRJNA1124845 |
| MeRIP-seq in cells (Raw sequencing data) | This paper | NCBI SRA: PRJNA1126087 |
| MeRIP-seq in methylated samples (Raw sequencing data) | This paper | NCBI SRA: PRJNA1126437 |
| eCLIP-seq (Raw sequencing data) | This paper | NCBI SRA: PRJNA1205343 |
| Origin data of WB about this paper | This paper | Mendeley Data, V1, doi: 10.17632/tk4392ff6c.1 |
| Origin analysis data about this paper | This paper | Mendeley Data, V1, doi: 10.17632/v3z2n8ksr7.1 |
| Experimental models: Cell lines | | |
| Human: HEK293T | This paper | N/A |
| Human: LoVo | This paper | N/A |
| Human: HCT8 | This paper | N/A |
| Human: HCT116 | This paper | N/A |
| Experimental models: Organisms/strains | | |
| BALB/c nude mice | Charles River | N/A |
| Oligonucleotides | | |
| HPD-qPCR-F: GGAGCCCTGGGTAGAGCAA | This paper | N/A |
| HPD-qPCR-R: CAAGAATTGGCCGATGTAGTTCA | This paper | N/A |
| PARP1-qPCR-F: CGGAGTCTTCGGATAAGCTCT | This paper | N/A |
| PARP1-qPCR-R: TTTCCATCAAACATGGGCGAC | This paper | N/A |
| SLC7A11-qPCR-F: TCTCCAAAGGAGGTTACCTGC | This paper | N/A |
| SLC7A11-qPCR-R: AGACTCCCCTCAGTAAAGTGAC | This paper | N/A |
| GPX4-qPCR-F: GAGGCAAGACCGAAGTAAACTAC | This paper | N/A |
| GPX4-qPCR-R: CCGAACTGGTTACACGGGAA | This paper | N/A |
| HMOX1-qPCR-F: AAGACTGCGTTCCTGCTCAAC | This paper | N/A |
| HMOX1-qPCR-R: AAAGCCCTACAGCAACTGTCG | This paper | N/A |
| FTL-qPCR-F: CAGCCTGGTCAATTTGTACCT | This paper | N/A |
| FTL-qPCR-R: GCCAATTCGCGGAAGAAGTG | This paper | N/A |
| SAT-qPCR-F: ACCCGTGGATTGGCAAGTTAT | This paper | N/A |
| SAT-qPCR-R: GCAACCTGGCTTAGATTCTTC | This paper | N/A |
| PRNP-qPCR-F: AGTCAGTGGAACAAGCCGAG | This paper | N/A |
| PRNP-qPCR-R: CTGCCGAAATGTATGATGGGC | This paper | N/A |
| RALBP1-qPCR-F: TGATGTTCCAAATCTCAAACCCA | This paper | N/A |
| RALBP1-qPCR-R: AGATGCCTTCACACTTCATGC | This paper | N/A |
| ZBTB1-qPCR-F: TGTGGCTCGAAATGGCAATGA | This paper | N/A |
| ZBTB1-qPCR-R: TTGACACACGTTCTTTTGGAGTA | This paper | N/A |
| STAR-qPCR-F: GGGAGTGGAACCCCAATGTC | This paper | N/A |
| STAR-qPCR-R: CCAGCTCGTGAGTAATGAATGT | This paper | N/A |
| METTL3-qPCR-F: TTGTCTCCAACCTTCCGTAGT | This paper | N/A |
| METTL3-qPCR-R: CCAGATCAGAGAGGTGGTGTAG | This paper | N/A |
| Actin-qPCR-F: GGAAATCGTGCGTGACAT | This paper | N/A |
| Actin-qPCR-R: TGCCAATGGTGATGACCT | This paper | N/A |
| Recombinant DNA | | |
| pcDNA3.1 | This paper | N/A |
| pcDNA3.1-Flag-HPD | This paper | N/A |
| pcDNA3.1-Flag-HPD-CMIm | This paper | N/A |
| pcDNA3.1-Flag-HPD-H183A | This paper | N/A |
| pcDNA3.1-Flag-HPD-H266A | This paper | N/A |
| pETM3C-Flag-HPD | This paper | N/A |
| pETM3C-Flag-HPD-CMIm | This paper | N/A |
| pETM3C-Flag-HPD-H183A | This paper | N/A |
| pETM3C-Flag-HPD-H266A | This paper | N/A |
| pcDNA3.1-Flag-METTL3 | This paper | N/A |
| pcDNA3.1-Flag-METTL3-CMIm | This paper | N/A |
| pcDNA3.1-Flag-METTL3-CMIIm | This paper | N/A |
| pETM3C-Flag-METTL3 | This paper | N/A |
| Software and algorithms | | |
| GraphPad Prism 8 | Graph- Pad | Graph- Pad |
| Adobe Illustrator | Adobe | Adobe |
| ImageJ | National Institutes of Health | National Institutes of Health |
| Leica Application Suite X -2.0.1 | Leica Microsystems | N/A |

**Supporting Figures and Legends**


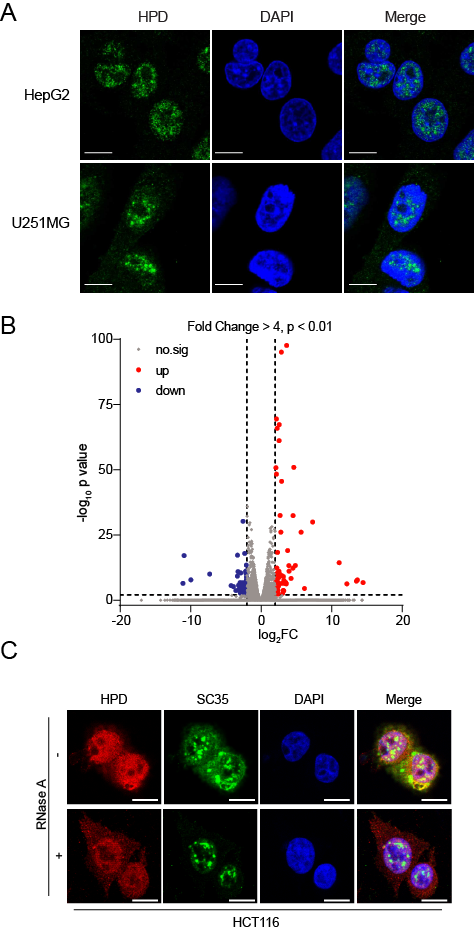


**Figure S1**

HPD is involved in RNA modification. A) Cellular localization of HPD in different cells from the Human Protein Atlas. Scar bars, 10 μm. B) Differentially expressed genes were analyzed by transcriptome sequencing in HPD knockdown cells. (shCtrl, n = 3; shHPD, n = 3). C) HCT116 cells treated with or without RNase A were stained for HPD, DAPI and SC35. Scar bars, 10 μm.


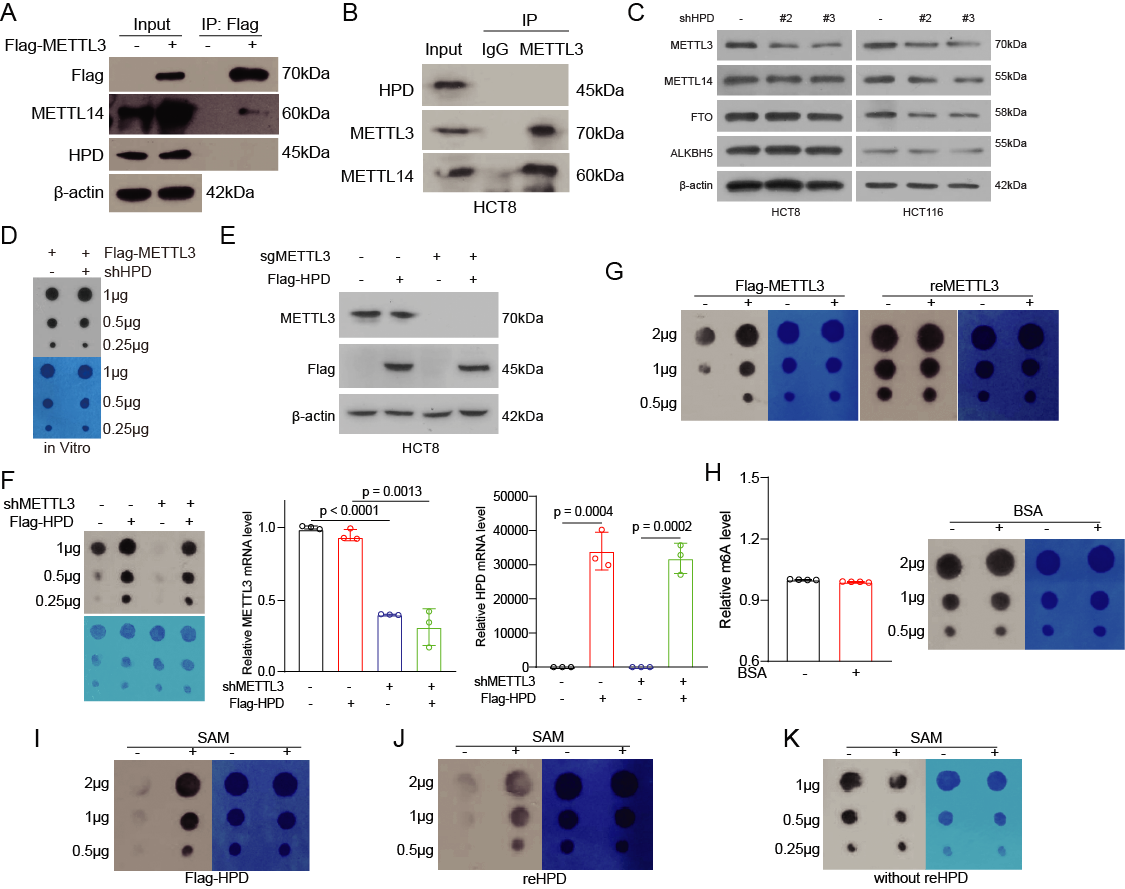


**Figure S2**

HPD regulates m^6^A modification independently of the methyltransferase METTL3. A) The interaction between HPD and m^6^A-related proteins was detected by co-immunoprecipitation assay in METTL3 overexpressing cells. B) The interaction between HPD and m^6^A-related proteins was detected by co-immunoprecipitation assay in CRC cells. C) The METTL3, METTL14, FTO and ALKBH5 proteins were detected in HPD knockdown cells. D) The m^6^A modification of total RNA was detected in CRC cells with HPD knockdown and re-overexpressed METTL3 by dot blot. E) The expression of METTL3 and HPD was detected in HCT8 cells with METTL3 knocout and re-overexpressed HPD. F) The m^6^A modification of total RNA was detected in HCT8 cells with METTL3 knockdown and re-overexpressed HPD by dot blot. G) The *in vitro* methylation assay was employed to assess the methyltransferase activity of Flag-METTL3 (from eukaryotic cell), reMETTL3 (from *E.coli*) and the m^6^A modification of total RNA was detected by dot blot. H) The *in vitro* methylation assay was employed to assess the methyltransferase activity of BSA. (Control, n = 4; BSA, n = 4). I-J) The *in vitro* methylation assay was employed for to assess the methyltransferase activity of proteins (Flag-HPD and reHPD) with or without SAM, and the m^6^A modification of total RNA was detected by dot blot. K) The *in vitro* methylation assay (removing reHPD) was employed to assess the methyltransferase activity with or without SAM, and the m^6^A modification of total RNA was detected by dot blot. Error bars in F and H, represent mean values ± SD, p values were determined by unpaired two-tailed Student’s t test of n = 3 independent biological experiments.


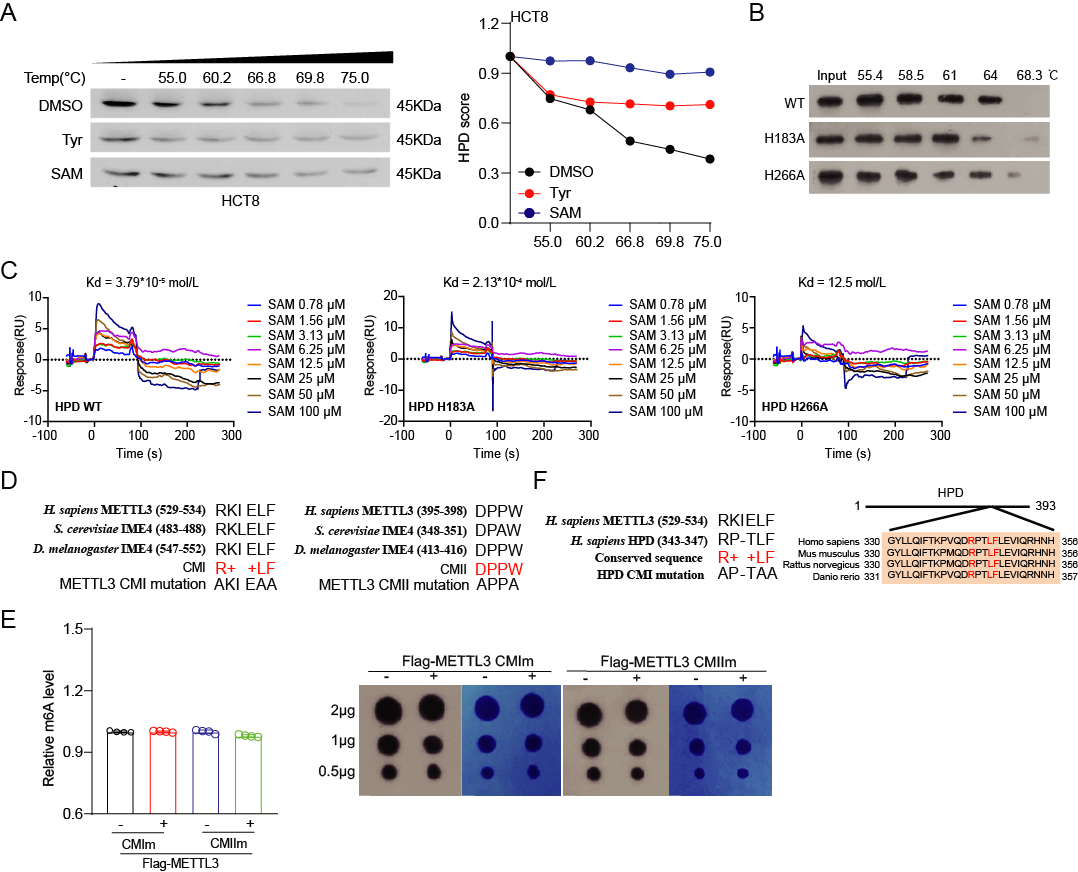


**Figure S3**

SAM is a cofactor for HPD methyltransferase. A) A thermol shift assay was performed to detect the binding between HPD and tyrosine or SAM. B) A thermol shift assay was performed to detect the binding between HPD (wildtype and mutant) and SAM. C) An SPR assay was performed to detect the binding between HPD (wildtype and mutant) and SAM. D) Conserved catalytic motifs and mutant forms in METTL3. E) An *in vitro* methylation assay was employed to assess the methyltransferase activity of Flag-METTL3 CMIm and CMIIm and the m^6^A modification of total RNA was detected by colorimetric assay and dot blot. F) Conserved catalytic motifs and mutant forms in HPD. Error bars in E, represent mean values ± SD, p values were determined by unpaired two-tailed Student’s t test of n = 4 technical replicates.


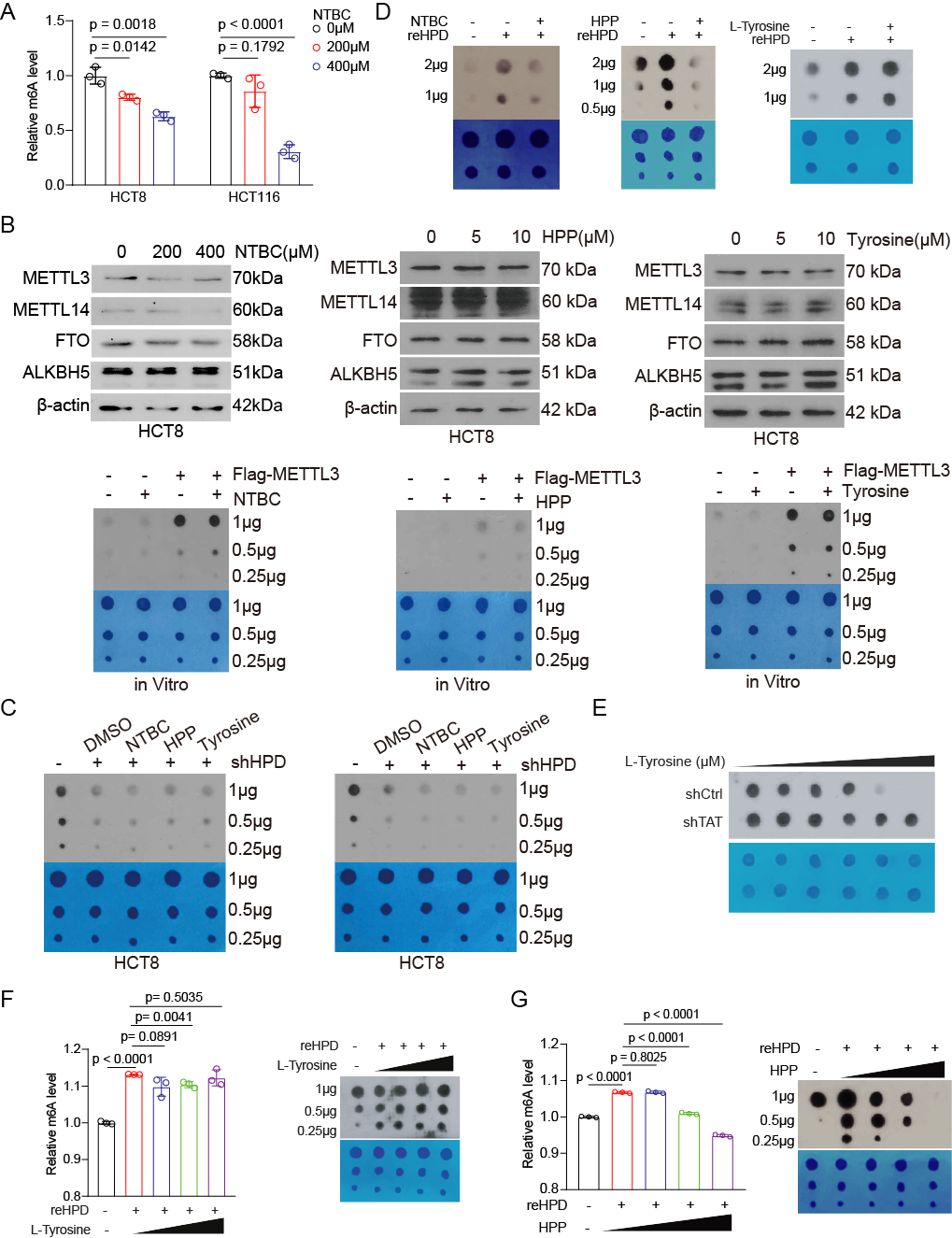


**Figure S4**

HPP, but not tyrosine, inhibits the methyltransferase activity of HPD. A) The m^6^A modification of mRNA was detected in cells treated with NTBC using LC/MS. B) The The METTL3, METTL14, FTO and ALKBH5 proteins were detected in NTBC, HPP or tyrosine treated cells. The m^6^A modification of total RNA was detected in HCT8 overexpressed METTL3 cells treated with NTBC, HPP or tyrosine by dot blot. C) The m^6^A modification of total RNA was detected in HPD knockdown cells treated with NTBC, HPP or tyrosine by dot blot. D) The *in vitro* methylation assay was employed to assess the methyltransferase activity of reHPD treated with different small molecules (HPP, NTBC and Tyrosine), and the m^6^A modification of total RNA was detected by dot blot. E) The m^6^A modification of total RNA was detected in HCT8 cells with TAT knockdown and treated with tyrosine by dot blot. F-G) The *in vitro* methylation assay was employed to assess the methyltransferase activity of reHPD treated with different concentrations of HPP or tyrosine. The m^6^A modification of total RNA was detected by dot blot and colorimetric assay. Error bars in A, F, and G, represent mean values ± SD, p values were determined by unpaired two-tailed Student’s t test of n = 3 technical replicates.


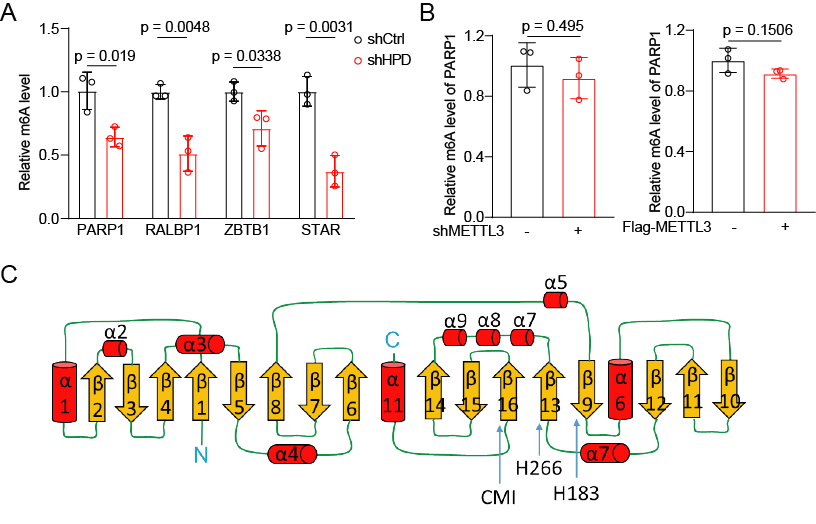


**Figure S5**

Identification of modification sites for HPD methyltransferases. A) The m^6^A modification was detected in knockdown HPD group and control group by MeRIP-qPCR. B) The m^6^A modification of PARP1 was detected in METTL3 knockdown cells and METTL3 overexpressing cells by MeRIP-qPCR. (C) Schematic representation of HPD structure. The arrows represent the β-strands and the cylinders representing the α-helices. Error bars in A and B, represent mean values ± SD, p values were determined by unpaired two-tailed Student’s t test of n = 3 technical replicates.


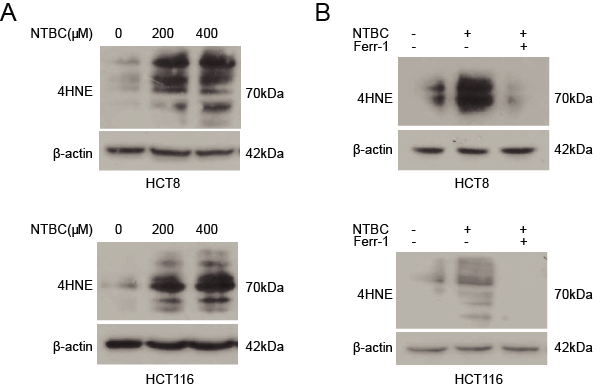


**Figure S6**

HPD regulates ferroptosis. A) The 4-HNE was detected in NTBC treated cells. B) The 4-HNE was detected in NTBC treated cells with or without Ferrostatin-1.


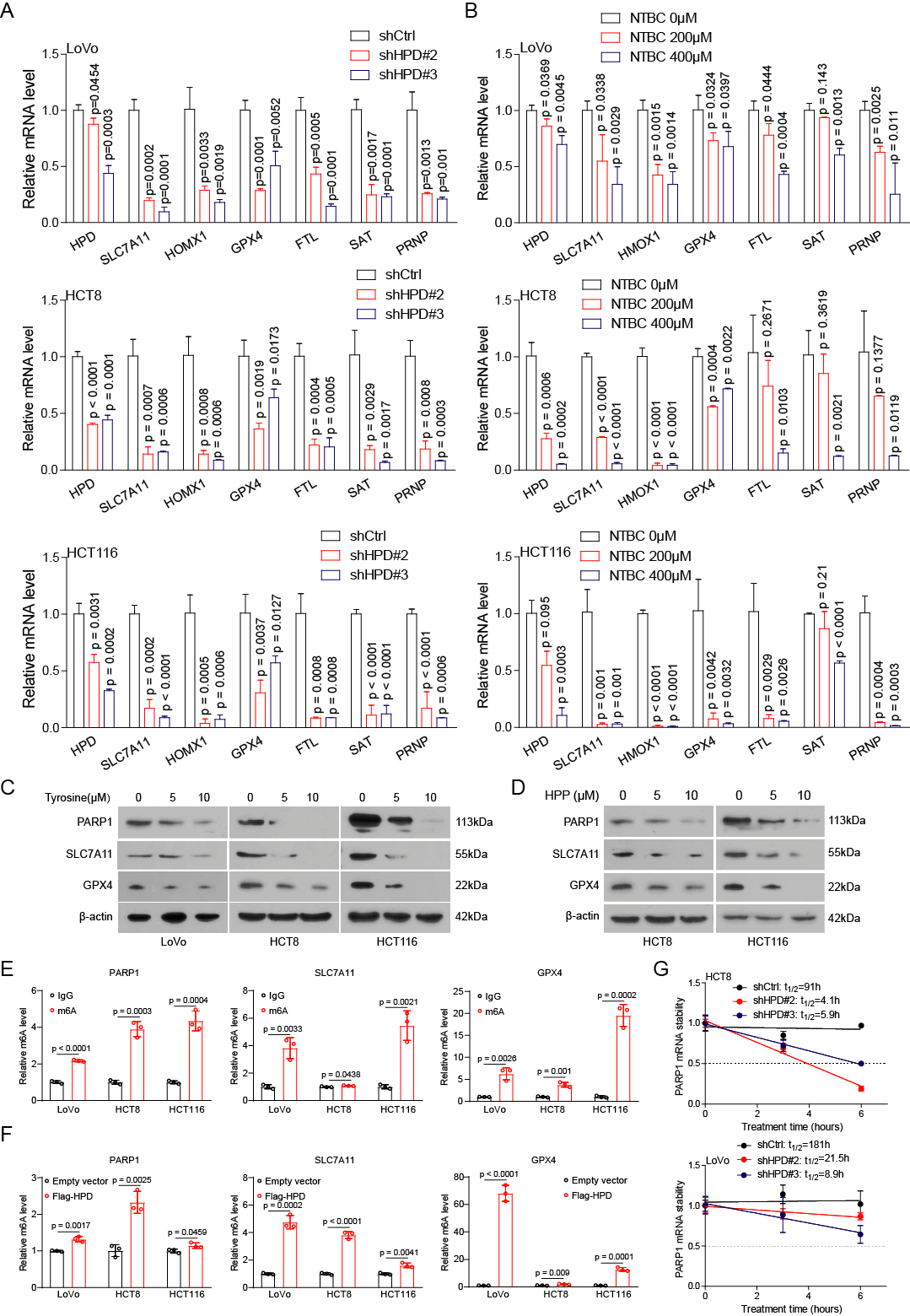


**Figure S7**

HPD regulates the expression of SLC7A11 and GPX4. A) The ferroptosis related genes were detected in HPD knockdown cells. B) The ferroptosis related genes were detected in NTBC treated cells. C-D) The PARP1, SLC7A11, and GPX4 protein were detected in Tyrosine or HPP treated cells. E) The m^6^A modification of the target gene was detected in different CRC cells by MeRIP-qPCR. F) The m^6^A modification was detected in HPD overexpressing cells and control group by MeRIP-qPCR. G) The PARP1 mRNA stability of target genes was detected in HPD knockdown and control group. Error bars in A, B, E, F, and G, represent mean values ± SD, p values were determined by unpaired two-tailed Student’s t test of n = 3 independent biological experiments.


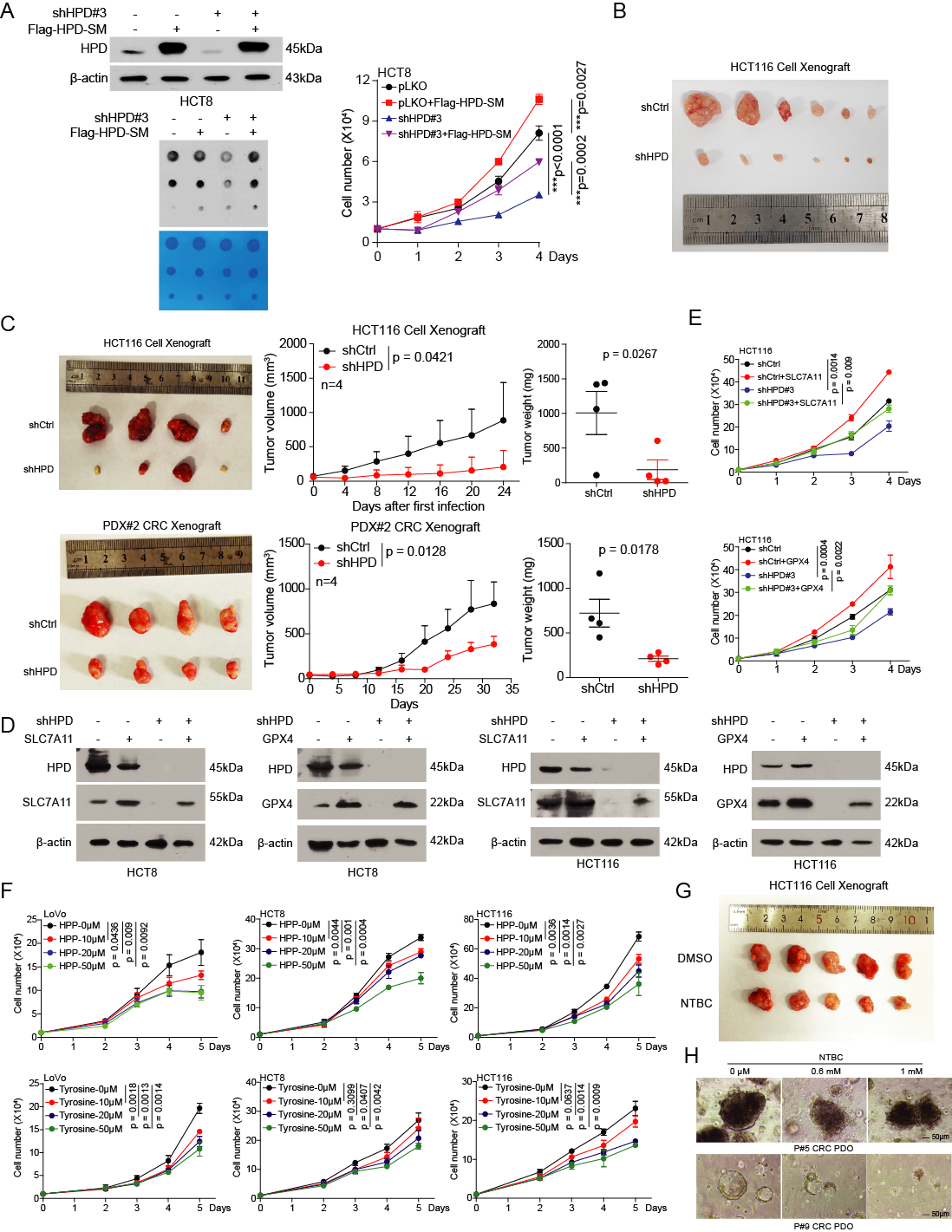


**Figure S8**

Knockdown of HPD and pharmacological inhibition suppress colorectal growth and proliferation. A) Cell proliferation and of m^6^A modification total RNA were determined in HPD knockdown cells with or without re-overexpressing shRNA-resistant HPD protein. B) The HCT116-CDX tumor models in nude mice showed tumor morphology (n = 6). C) Tumor growth rates and tumor weights were recorded in the CDX and PDX model. The CDX and PDX tumor models in nude mice showed tumor morphology (n = 4). D-E) Cell proliferation was determined in HPD knockdown cells with or without re-overexpressing SLC7A11/GPX4. F) Ccell proliferation was determined in Tyrosine or HPP treated cells. G) The HCT116-CDX models treated with NTBC in nude mice showed tumor morphology. H) The CRC organoid growth was determined with the treatment of NTBC. Error bars in A, C, E and F, represent mean values ± SD, p values were determined by unpaired two-tailed Student’s t test of n = 3 independent biological experiments.


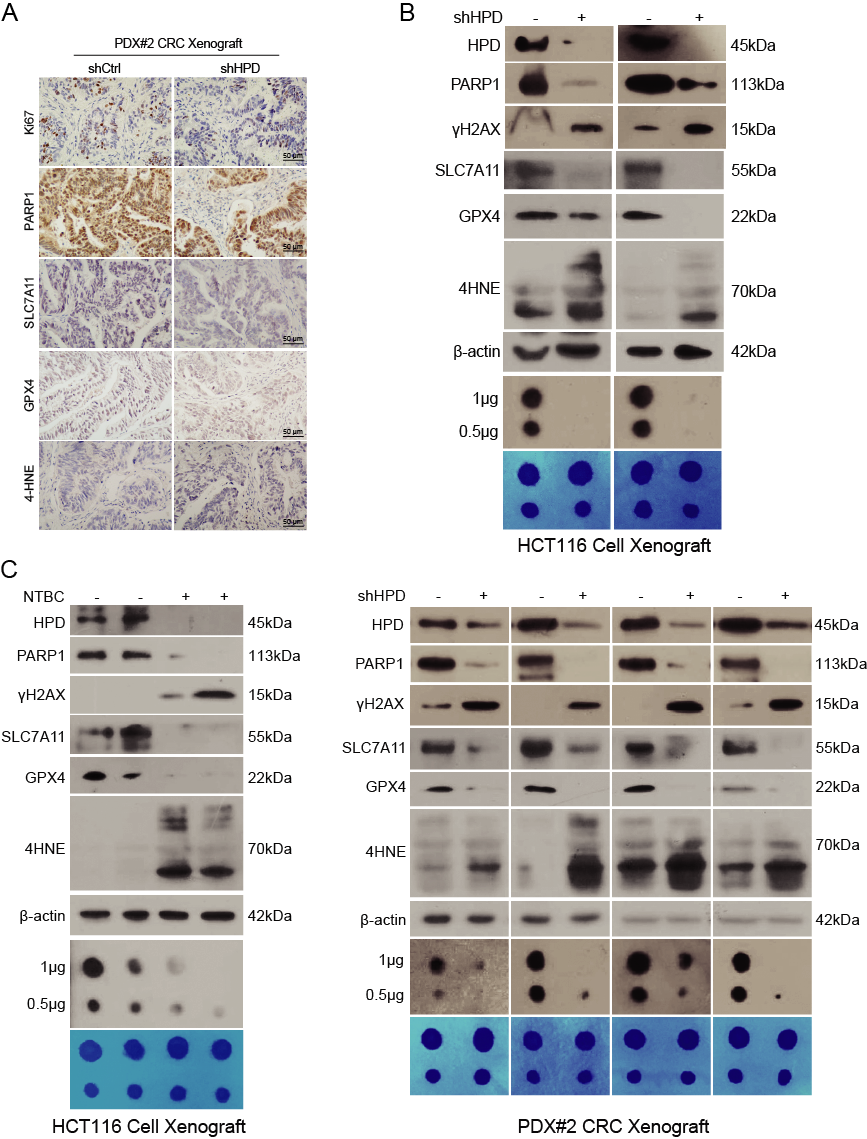


**Figure S9**

HPD regulates DNA damage and ferroptosis in CRC tumors. A-C) The target proteins, DNA damage markers and ferroptosis markers were detected by immunoblotting and immunohistochemistry in the tumor tissues of the HCT116-CDX and PDX model.

**Original unaltered images of western blots**


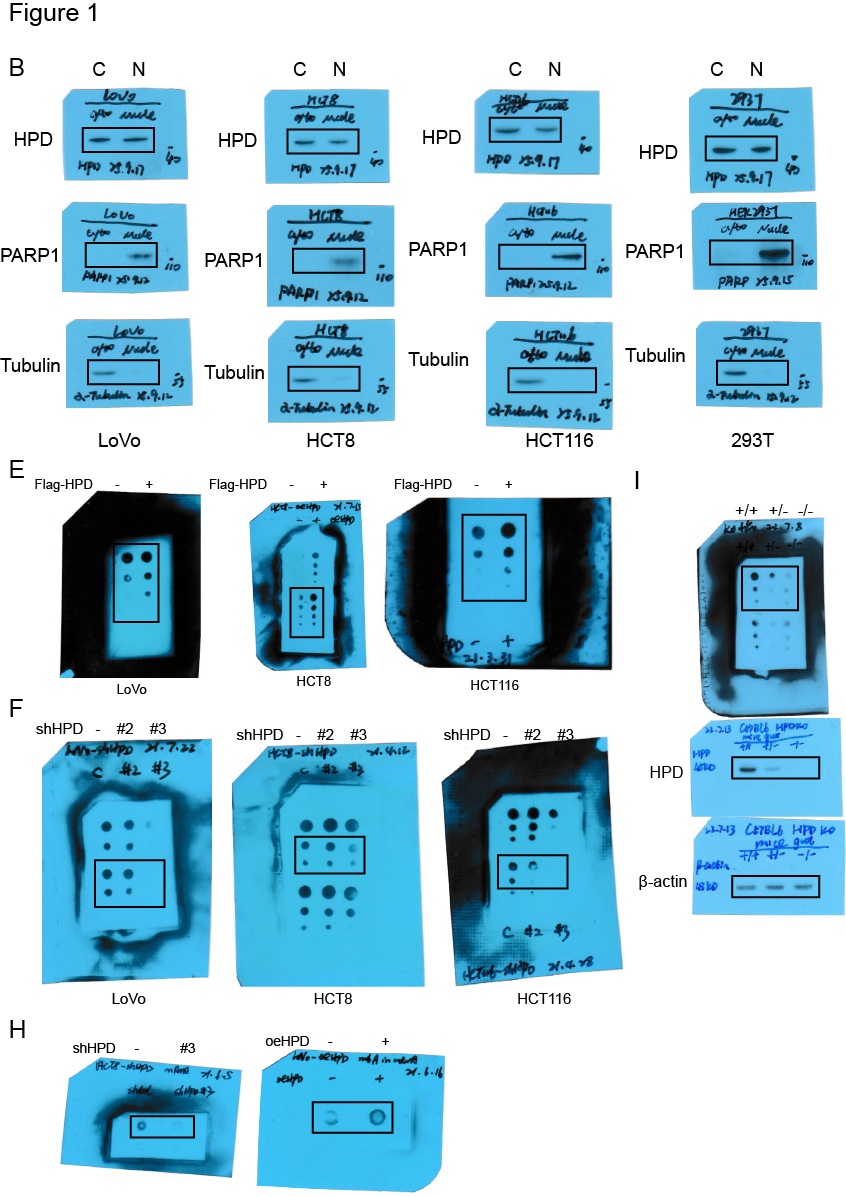


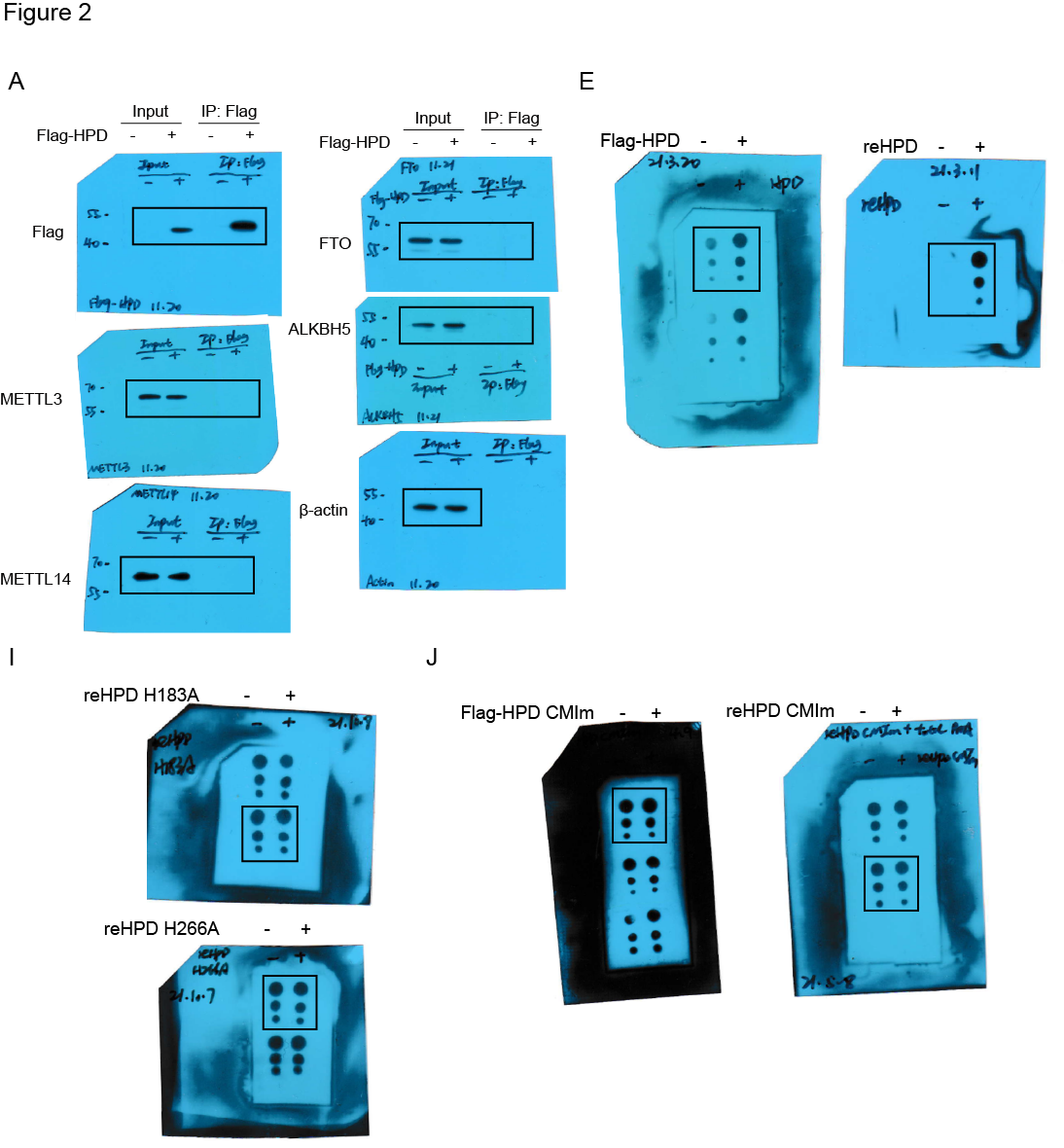


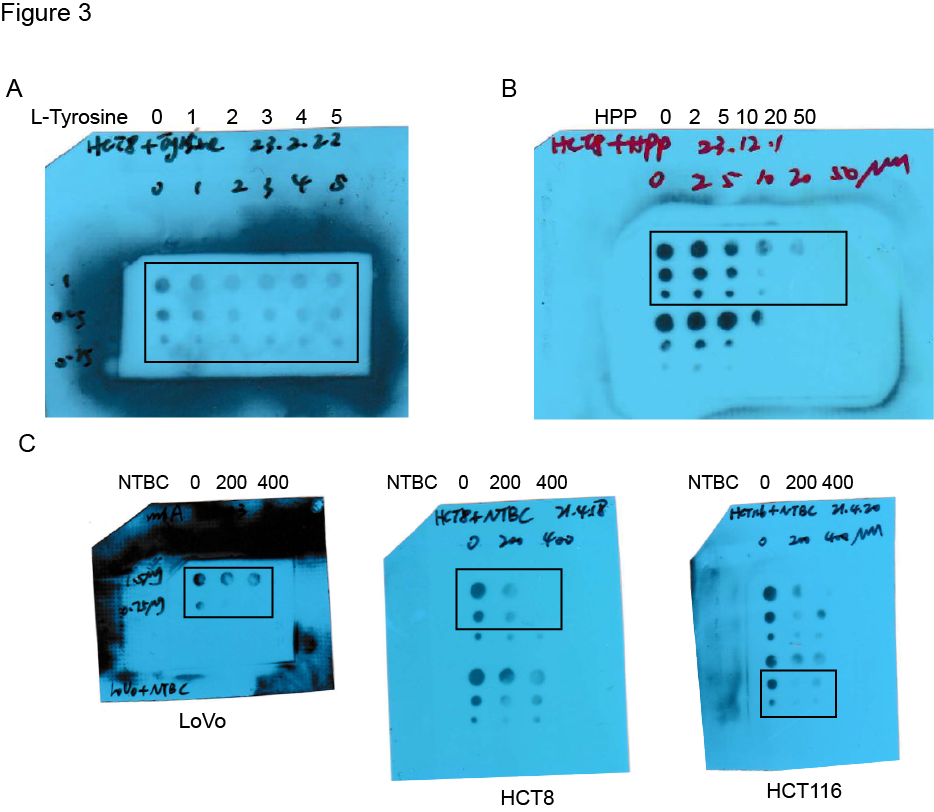


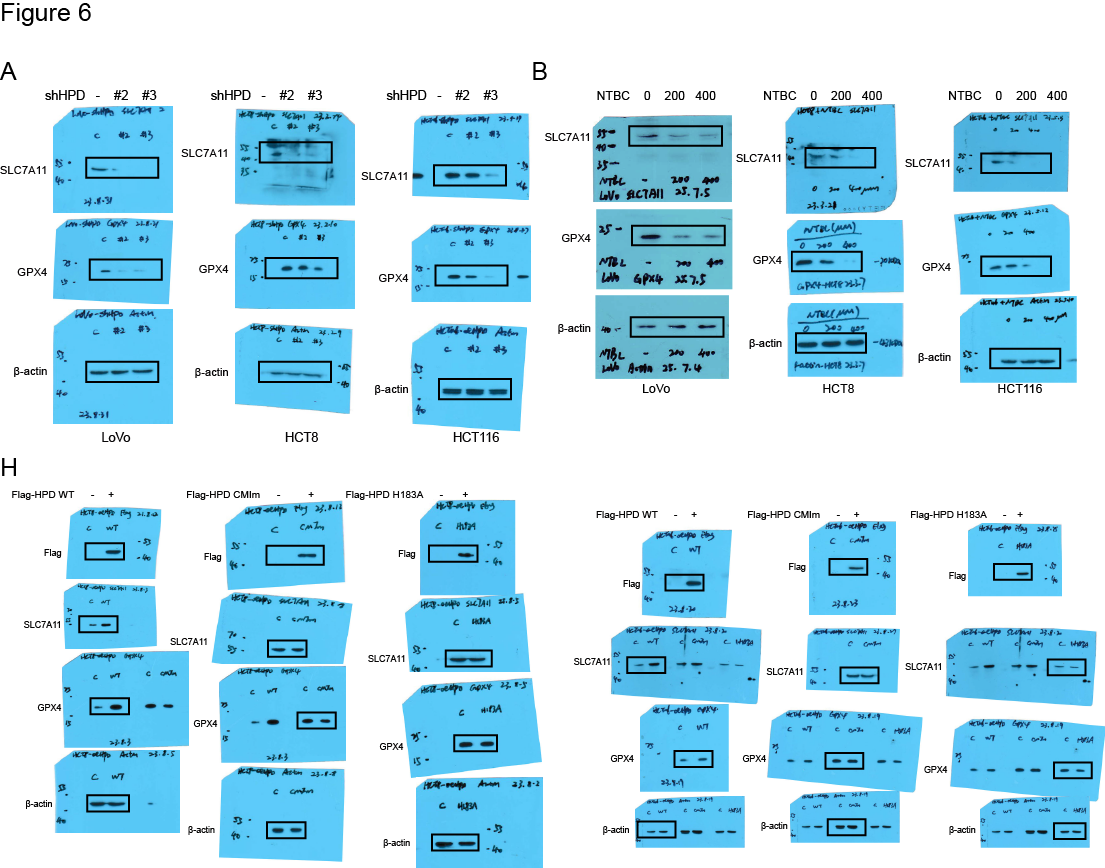


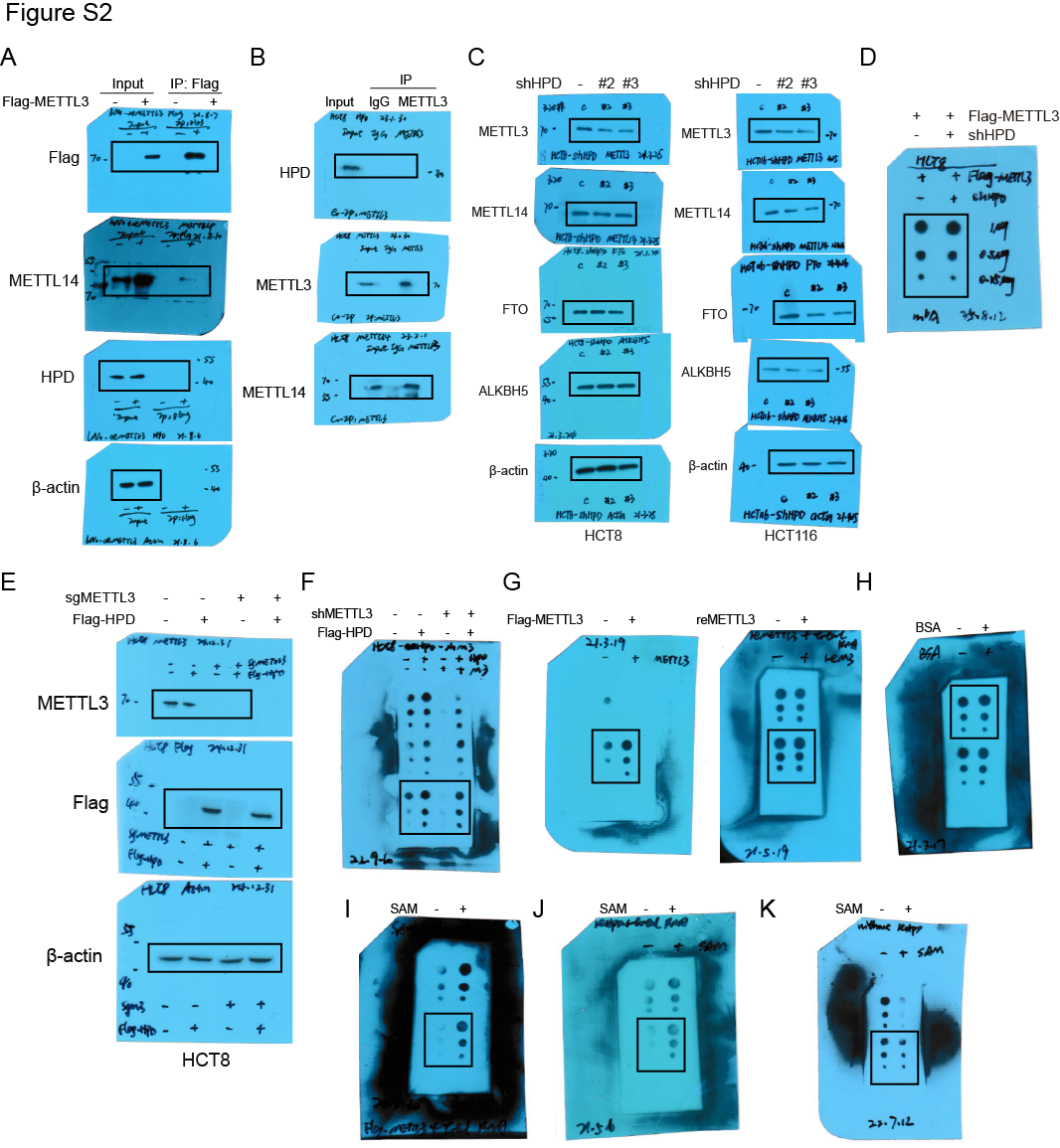


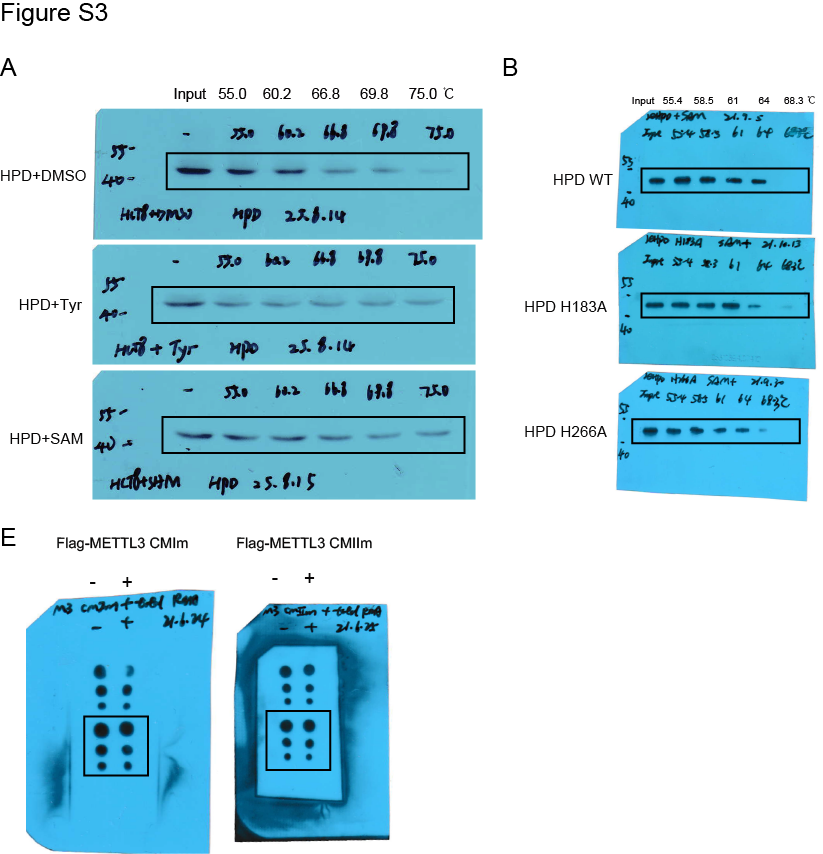


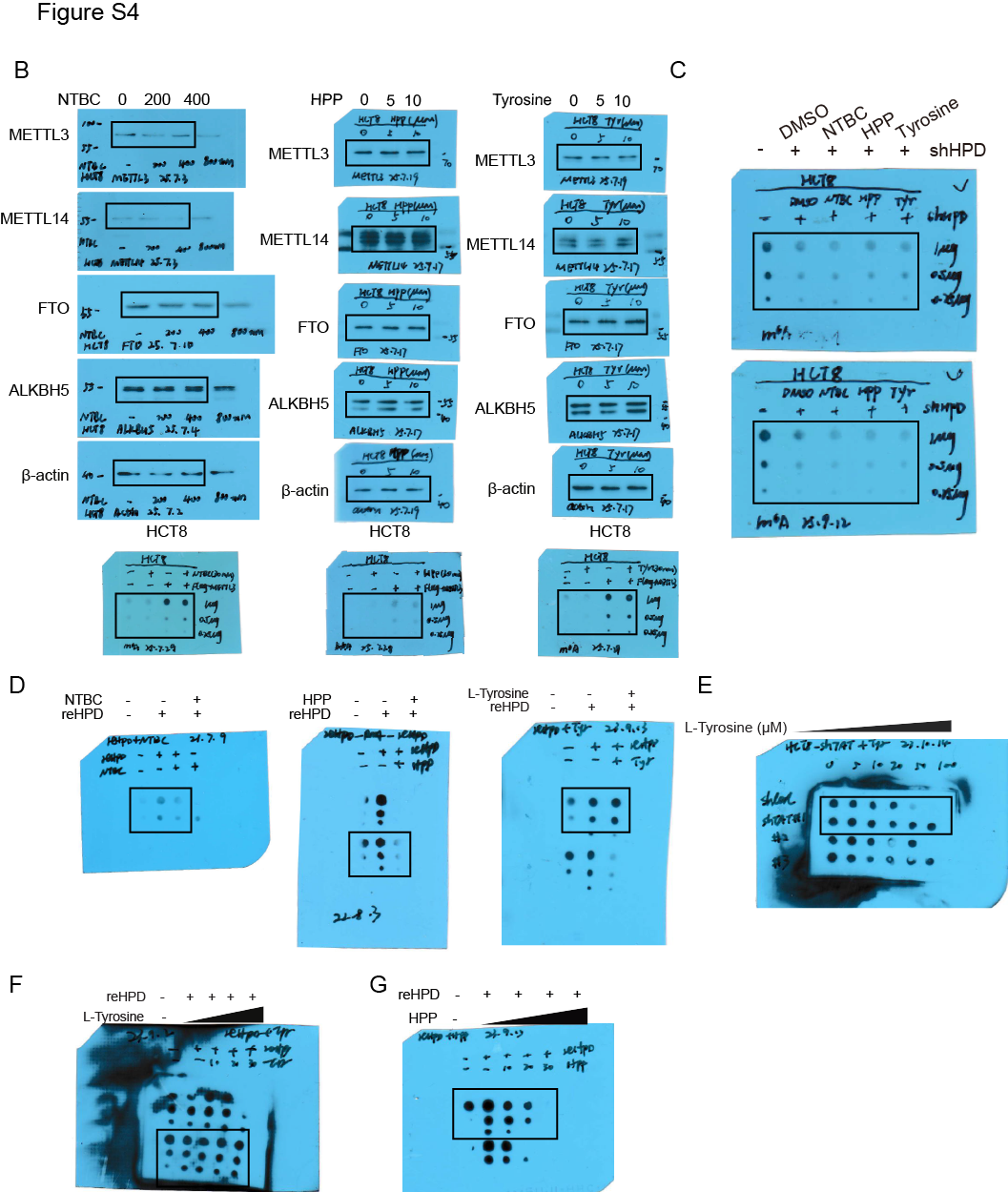


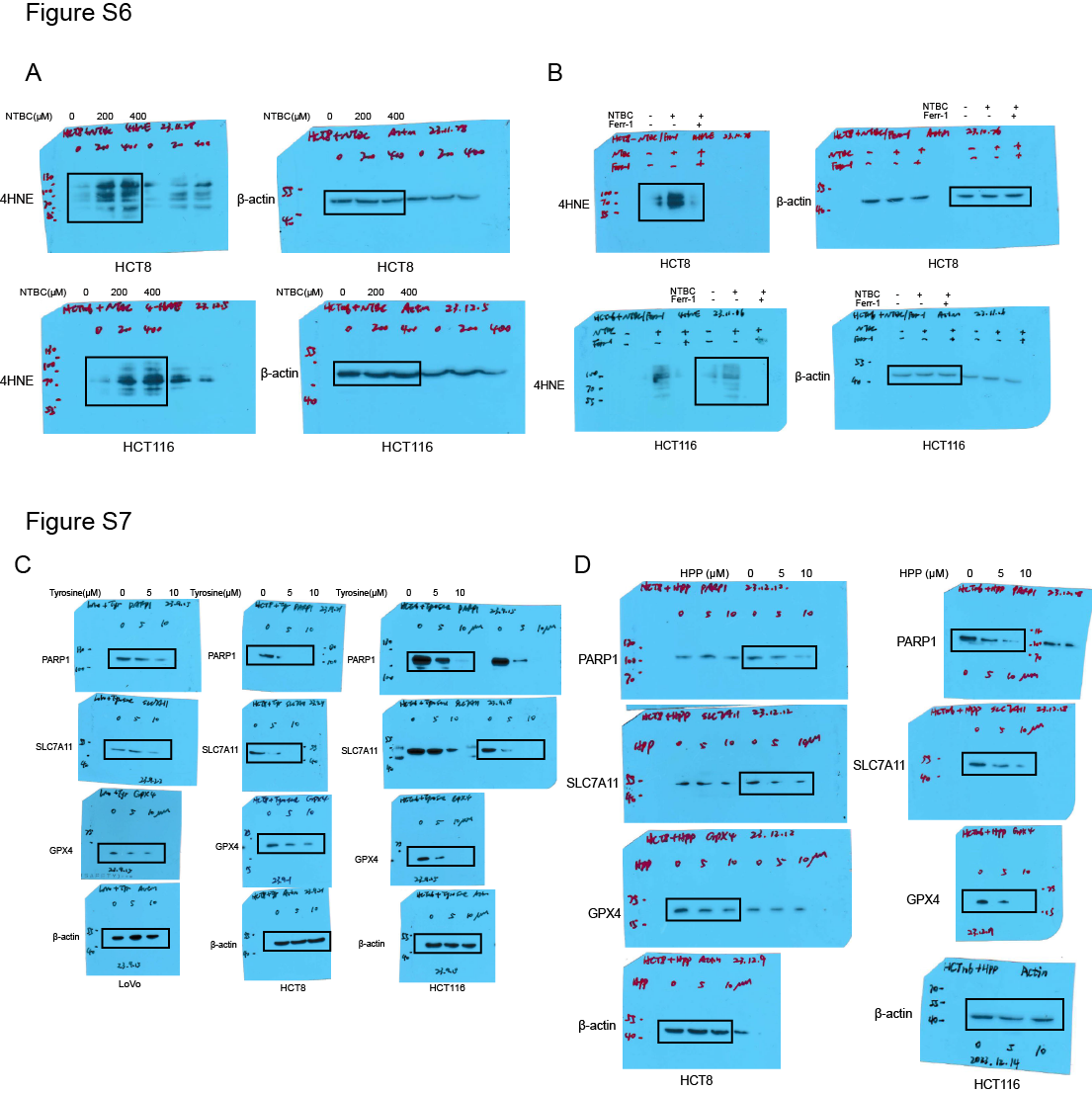


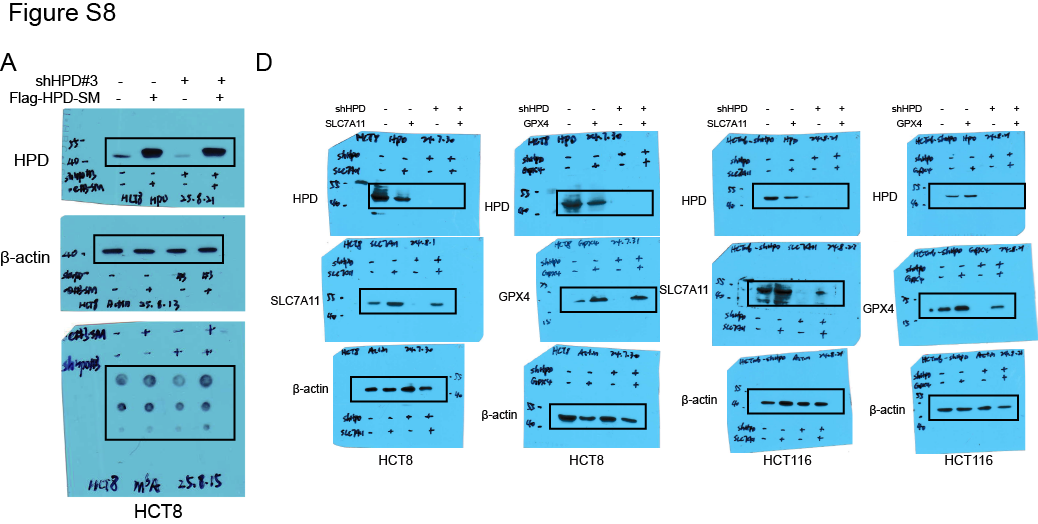


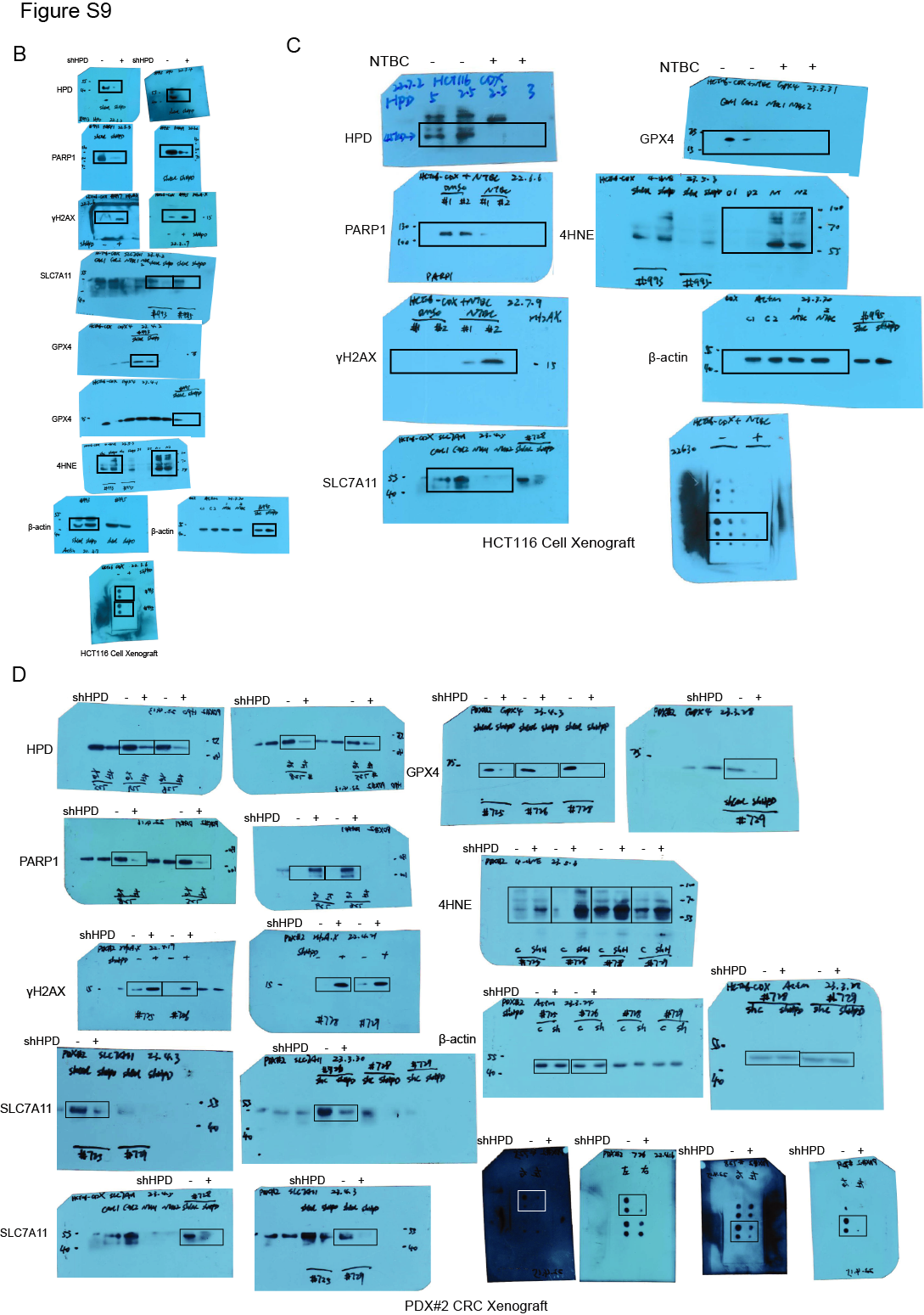

Supplement: Supplementary file 1 — Supporting Information [file ADVS-13-e08541-s001.docx]
